# Identification of expression quantitative trait loci associated with schizophrenia and affective disorders in normal brain tissue

**DOI:** 10.1371/journal.pgen.1007607

**Published:** 2018-08-24

**Authors:** Oneil G. Bhalala, Artika P. Nath, UK Brain Expression Consortium, Michael Inouye, Christopher R. Sibley

**Affiliations:** 1 Systems Genomics Lab, Baker Heart and Diabetes Institute, Melbourne, Victoria, Australia; 2 The Royal Melbourne Hospital, Melbourne Health, Parkville, Victoria, Australia; 3 Department of Microbiology and Immunology, The Peter Doherty Institute, University of Melbourne, Parkville, Victoria, Australia; 4 Cambridge Baker Systems Genomics Initiative, Baker Heart & Diabetes Institute, Melbourne, Victoria, Australia; 5 Cambridge Baker Systems Genomics Initiative, Department of Public Health and Primary Care, University of Cambridge, Cambridge, United Kingdom; 6 Department of Clinical Pathology, The University of Melbourne, Parkville, Victoria, Australia; 7 Department of Public Health and Primary Care, University of Cambridge, Cambridge, United Kingdom; 8 The Alan Turing Institute, British Library, London, United Kingdom; 9 Department of Molecular Neuroscience, University College London Institute of Neurology, Russell Square House, Russell Square, London, United Kingdom; 10 Department of Medicine, Division of Brain Sciences, Imperial College London, Burlington Danes, London, United Kingdom; Mount Sinai School of Medicine, UNITED STATES

## Abstract

Schizophrenia and the affective disorders, here comprising bipolar disorder and major depressive disorder, are psychiatric illnesses that lead to significant morbidity and mortality worldwide. Whilst understanding of their pathobiology remains limited, large case-control studies have recently identified single nucleotide polymorphisms (SNPs) associated with these disorders. However, discerning the functional effects of these SNPs has been difficult as the associated causal genes are unknown. Here we evaluated whether schizophrenia and affective disorder associated-SNPs are correlated with gene expression within human brain tissue. Specifically, to identify expression quantitative trait loci (eQTLs), we leveraged disorder-associated SNPs identified from 11 genome-wide association studies with gene expression levels in post-mortem, neurologically-normal tissue from two independent human brain tissue expression datasets (UK Brain Expression Consortium (UKBEC) and Genotype-Tissue Expression (GTEx)). Utilizing stringent multi-region meta-analyses, we identified 2,224 *cis*-eQTLs associated with expression of 40 genes, including 11 non-coding RNAs. One *cis*-eQTL, rs16969968, results in a functionally disruptive missense mutation in *CHRNA5*, a schizophrenia-implicated gene. Importantly, comparing across tissues, we find that blood eQTLs capture < 10% of brain *cis*-eQTLs. Contrastingly, > 30% of brain-associated eQTLs are significant in tibial nerve. This study identifies putatively causal genes whose expression in region-specific tissue may contribute to the risk of schizophrenia and affective disorders.

## Author summary

An estimated 21 million people live worldwide with schizophrenia, 60 million with bipolar disorder, and 400 million with major depressive disorder. Recent genome-wide association studies have shed light on the genetic variants linked to these disorders, and increasing evidence suggests that their genetic architectures may overlap. However, understanding the roles of these variants in disease biology remains limited. Here we questioned whether genetic variation associated with these disorders is correlated with the expression of genes that are proximally located within the genome. Importantly, we evaluate this in two large and independent human brain tissue datasets. We subsequently identify, with high confidence, >2,200 disease-associated variants as putative regulators of expression for nearby genes. The identification of these regulated genes provides new insights into disease biology and will help prioritise associations for future mechanistic follow-up studies.

## Introduction

Schizophrenia and affective disorders, comprising bipolar disorder and major depressive disorder, constitute a significant global burden of disease. Worldwide it is estimated that more than 21 million individuals are living with schizophrenia, 60 million with bipolar disorder and over 400 million with major depressive disorder [1]. The consequences are staggering as evidenced by the fact these three diseases, which usually emerge in early-adulthood, accounted for over 90 million disability-adjusted life years in 2010 [2].

Ineffective management, which contributes to the enormous disease burden, is largely due to our lack of understanding about the pathobiology underlying these disorders. Family and twin studies have estimated heritability to be between 70–80% for schizophrenia and bipolar disorder and up to 40% for major depressive disorder [3]. This has prompted the establishment of genome-wide association studies (GWAS) to identify genetic variants associated with these disorders. Recent and large-scale GWAS such as those organized the Psychiatric Genomics Consortium (PGC), CONVERGE Consortium and 23andMe, have been published for schizophrenia [4–7], bipolar disorder [8–10], major depressive disorder [11–13] as well as for a multiple-disorder analysis [14]. Results from these studies have suggested that schizophrenia, bipolar disorder and major depressive disorder may share common genetic architecture [15, 16].

While GWAS have identified numerous loci associated with human diseases [17, 18], understanding their roles in disease biology remains limited. Expression quantitative trait loci (eQTLs) are genetic variants that affect gene expression levels and may offer insights into mechanisms contributing to health and disease [19–21]. An eQTL can act in *cis*, meaning that the variant is associated with expression of a gene within 1Mb, or in *trans*, with the variant located outside of this window. Studies have leveraged GWAS data, particularly that of single nucleotide polymorphisms (SNPs), to identify eQTLs [22, 23]. Moreover, it is becoming evident that disease-associated variants are enriched for eQTLs [14, 24]. Hence, eQTL studies based on disease-GWAS may implicate important molecular processes underlying pathobiology.

In this study, we therefore sought to identify eQTLs associated with schizophrenia and affective disorders in neurologically-normal post-mortem brain tissue. By leveraging gene expression data from UK Brain Expression Consortium (UKBEC) [25] and NIH Genotype-Tissue Expression (GTEx) [26] and performing multi-region analyses [27], we identified *cis*-eQTLs that were pervasive across various brain regions and determined the extent to which these overlapped with those detected in more accessible tissue including blood and peripheral nervous system. Results reported here may help prioritize future studies of GWAS SNPs associated with these disorders.

## Methods

### Collation of analysis-SNPs from GWAS data

**Fig 1:**
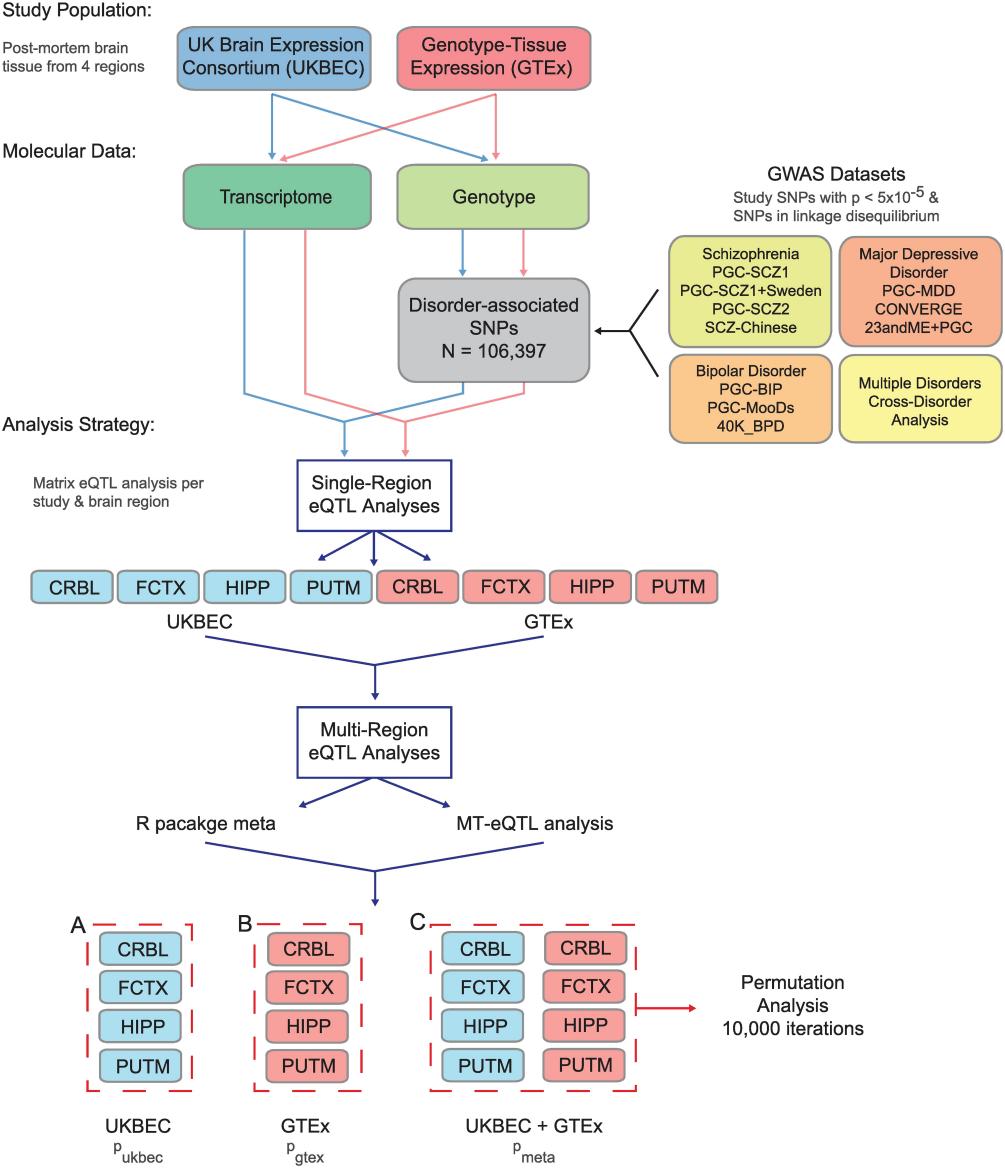
Study design for identification of multi-region brain eQTLs. Two studies with genotype and transcriptomic data (UKBEC and GTEx) were analysed for eQTLs based on SNPs that were found to be associated with schizophrenia, bipolar disorder and major depressive disorder through 11 GWAS studies. UKBEC consisted of samples from neurologically-normal individuals; samples from individuals with neurological disease (see Methods) were removed from GTEx prior to analyses. Single-region eQTL analyses were performed for each study and brain region using Matrix eQTL. Next, multi-region eQTL analyses were performed using two methods: (1) the R package meta and (2), the MT-eQTL analysis package. Intersection of these results yielded the list of multi-region meta-analysis eQTLs for UKBEC (p_ukbec_), GTEx (p_gtex_) and UKBEC+GTEx (p_meta_). Results from UKBEC+GTEx were further analysed in the study, including permutation analysis. *CRBL*, cerebellum; *FCTX*, frontal cortex; *HIPP*, hippocampus; *PUTM*, putamen.

Publicly available GWAS data from the 11 most-recent and largest studies (those with >10,000 cases and controls) related to schizophrenia, bipolar disorder and major depressive disorder were selected for analysis (Fig 1). SNPs were collated from the following studies: PGC-SCZ1 [4], PGC-SCZ1+Sweden [5], PGC-SCZ2 [6] and SCZ-Chinese [7] for schizophrenia; PGC-BIP [8], PGC-MooDs [9] and 40K_BPD [10] for bipolar disorder; PGC-MDD [11], CONVERGE [12] and 23andMe [13] for major depressive disorder, and PGC-Cross Disorder Analysis [14] for multiple disorders (schizophrenia, bipolar disorder, major depressive disorder, autism spectrum disorder and attention-deficit hyperactivity disorder). Of note, many of these studies included samples that were analysed in previous studies (S1 Table). We included overlapping studies to maximize the number of disorder-associated SNPs in our analysis as there may be loci identified in one study but not in another.

Guided by the NHGRI-EBI GWAS catalogue [17, 28], we included disease-associated SNPs with a study p-value < 5 x 10^−5^ in our analysis in order to also capture those SNPs with suggestive associations. For SNPs from all studies except PGC-SCZ1+Sweden, PGC-SCZ2 and 23andMe, we also obtained SNPs that are in moderate-high linkage disequilibrium (LD, R^2^ ≥ 0.5) with the study-SNPs [29] using the web-based tool rAggr (http://raggr.usc.edu/). LD-analysis settings were as follows: CEU population from 1000 Genomes Phase 3 October 2014 release, build hg19, minimum minor allele frequency = 0.001, maximum distance = 500kb, maximum Mendelian errors = 1, cut-off for Hardy-Weinberg p-value = 0, and minimum genotype = 75%. Study-SNPs from PGC-BIP, PGC-MDD and PGC-Cross Disorder Analysis were lifted from hg18 to hg19 prior to LD-analysis. SNPs in LD with study-SNPs from PGC-SCZ1+Sweden, PGC-SCZ2 and 23andMe were not obtained as these studies had >5,000 study-SNPs and were not LD-pruned. A total of 106,397 analysis-SNPs (from autosomal and X chromosomes) resulted from collation of the 11 studies (Fig 1, S1 Table).

### Genotype and expression data

Genotype and gene expression data were obtained from UKBEC [25]. These data contained samples from 134 neurologically-normal individuals from the following brain regions: cerebellum, frontal cortex, hippocampus and putamen (see S2 Table for sample sizes per region). UKBEC study brain regions not overlapping with GTEx brain regions were not included in this study. Genotype processing is previously described [25]. Briefly, these data included ~5.88 million imputed (1000 Genome, March 2012 release) and typed SNPs. Raw expression data from Affymetrix Human Exon 1.0 ST microarrays were processed as described previously with minor modifications. Specifically, all ~5 million probes were initially re-mapped to Ensembl v75 annotations using BioMart. Only probe sets containing three or more probes free of the ‘polymorphism-in-probe’ problem were used for subsequent analysis [30]. Following robust multi-array average (RMA) normalisation and background filtering, we calculated gene-level estimates by taking the winsorised mean of all probe sets for a given gene. Prior to eQTL detection, these were covariate-corrected for sex, cause of death, post-mortem interval, RNA integrity number (RIN) and study group using a linear regression model.

Data from GTEx [26] (dbGaP accession phs000424.v6.p1) were also included in our study. The following brain regions were analysed: cerebellum, frontal cortex (BA9), hippocampus and putamen (see S2 Table for sample sizes per region). GTEx study brain regions not overlapping with UKBEC brain regions were not included in this study. Whole blood and tibial nerve served as comparisons to brain tissues. Genotype and expression processing is described at http://gtexportal.org. Briefly, the dataset contained ~11.55 million imputed (1000 Genomes, August 2012 release) and typed autosomal variants and ~26,000 transcripts. To make this dataset conform with UKBEC, we excluded GTEx individuals identified as having neurological diseases based on description of their comorbidities/cause of death or inclusion of certain variables (see S2 Table for variable list). Prior to eQTL detection, gene expression was covariate-corrected for first three genotyping principal components (PCs), array platform, sex and probabilistic estimation of expression residuals (PEER) factors using a linear regression model.

### Single-region eQTL analyses

The R package Matrix eQTL [31] was used to identify eQTLs. *Cis*-eQTLs were defined as SNPs within 1Mb of the transcription start site; those SNPs outside this region, i.e. *trans*-eQTLs, were not considered in this analysis. Study genotype data were limited to those that were present in the set of analysis-SNPs described above. To minimize eQTL signals being called as significant when only present as a strong effect in a few individuals, analysis-SNPs that had a minor allele frequency < 5% within the analysis population were excluded. Gene expression input was based on expression residuals from linear regression of gene expression and genotype. *cis*-eQTL analysis was performed independently for each region per study (S5 and S6 Figs). eQTL (beta) effect sizes are given as standardised expression units (EU) per allele and each eQTL is a unique SNP-gene pair. We used the stringent Bonferroni correction method to set the critical p-value threshold per brain region and gene expression database combination analysed; hence each combination had its own unique p-value threshold (S3 Table). For completeness, in S3 Table, we also included the number of eQTLs with a False Discovery Rate (FDR) < 0.05.

### Multi-region eQTL analyses

To determine which eQTLs were present in multiple tissues, we performed two separate approaches that were then intersected. First, and using single-region eQTL results, we performed a meta-analysis of test statistics (eQTL effect size estimates and standard errors calculated by Matrix eQTL) using the metagen function in the R package meta with a random-effects model. FDR was calculated using R p.adjust with the Benjamini-Hochberg method. Three groups of meta-analyses were performed (Fig 1): A) four regions in UKBEC (p-value denoted as p_ukbec_); B) four regions in GTEx (p_gtex_); and C) of eight regions between UKBEC and GTEx (p_meta_). The first ten principal components of the UKBEC-GTEx meta-analysis for each of the overlapping regions are shown in S1–S4 Figs. As above, we used the stringent Bonferroni correction method to set the critical p-value threshold per brain region and gene expression database combination being analysed (S4 Table). For completeness, in S4 Table, we also include the number of eQTLs with an FDR < 0.05.

Second, we utilized a hierarchical Bayesian model for multi-region eQTL analysis (MT-eQTL) [27]. This model incorporates variation patterns of the presence/absence of eQTLs as well as their effect size heterogeneity across tissues. MT-eQTL indicates in which tissue(s) a gene-SNP pair is expected to be an eQTL (*isEQTL* variable). We similarly applied this approach across three groups (Fig 1): four regions in UKBEC; B) four regions in GTEx; and C) of eight regions between UKBEC and GTEx. An eQTL was deemed probable in each analysis if *isEQTL* = 1 in all tissues tested (ie all four tissues in UKBEC and GTEx, separately and all eight tissues in UKBEC and GTEx combined, S4 Table).

Through intersection of both analyses, we defined an eQTL as high confidence if (1), the meta-analysis p-value was less than the p-value threshold set by the Bonferroni correction (S4 Table), and (2), it was determined to be an eQTL in all tissues assessed using MT-eQTL (S4–S7 Tables, S7 Fig). To further validate this approach, permutation analysis was used to test the robustness of eQTLs identified in the multi-region meta-analysis of overlapping regions in UKBEC and GTEx datasets (Fig 1, analysis C). 10,000 iterations were performed for each region-study combination per high-confidence eQTL by shuffling sample IDs in the gene expression file prior to eQTL analysis. Meta-analysis of each permuted iteration per combination yielded a meta p-value. Permuted p-value was calculated as the number of meta p-values equal to or less than the nominal p-value (p_meta_), divided by the number of iterations (10,000). Those eQTLs with permuted p-values less than 5 x 10^−6^ (0.05/10000) surpassed permutation testing, thus revealing that all high-confidence eQTLs reached this stringent threshold. The aim of this rigorous and stringent thresholding was to identify disease-associated eQTLs across multiple brain regions with high confidence.

### Overlap with other datasets

*Cis*-eQTLs identified in the multi-region meta-analysis above (Fig 1, analysis C) were assessed for overlap with *cis*-eQTLs from GTEx samples of whole blood and tibial nerve tissue (calculated using Matrix eQTL as described above, S2 Table), as well as those downloaded from the Blood eQTL Browser (http://genenetwork.nl/bloodeqtlbrowser/) [32]. eQTL beta effect sizes for the GTEx samples and meta-analysis were converted to z-scores using the R scale with center = TRUE and scale = TRUE for effect size comparison; for eQTLs from the Blood eQTL Browser, the “OverallZscores” variable was used.

We also accessed for overlap with two recently published eQTL studies involving human dorsolateral prefrontal cortex tissue. The Religious Orders Study and Memory and Ageing Project (ROSMAP) study [33] analysed QTL data from 411 older individuals and resulting *cis-*eQTL associations were downloaded from the Brain xQTLServe (http://mostafavilab.stat.ubc.ca/xQTLServe/). *cis-*eQTLs with FDR < 0.05 from the CommonMind Consortium (CMC) case-control study [34] of over 250 individuals with schizophrenia were obtained from the CMC Knowledge Portal (https://www.synapse.org/#!Synapse:syn2759792/wiki/69613).

Replication rates of this study’s brain tissues eQTL analysis with other tissues and databases were assessed using the π_1_ statistic [35], which estimates the proportion of non-null hypotheses. For eQTL overlap between multi-region meta-analysis (Fig 1, analysis C) and GTEx whole blood, GTEx tibial nerve and ROSMAP [33], the R package qvalue was utilised. As Blood eQTL [32] and CMC [34] data only reported limited eQTLs, available FDR values were used to estimate π_1_.

### Determination of colocalisation between GWAS and eQTL signals

Bayesian colocalisation analysis was conducted to assess the extent of overlap between eQTL (related to the PGC-SCZ2 [6] study) and GWAS signals using the R package Coloc [36]. Summary statistics of all SNPs (regardless of GWAS p-value) within 200kb of the lead GWAS SNP and common in both the GWAS and eQTL studies were inputted into Coloc, which was run with default parameter settings [36]. Regions showing evidence of colocalisation between the GWAS and eQTL signals were identified utilizing pre-defined thresholds [37]: PP3 (posterior probability that there exist two distinct causal variants, one for each trait) + PP4 (posterior probability that these exists a single causal variant common to both traits) ≥ 0.80 and PP4/PP3 ≥ 3.

## Results

### Identification of disease-associated eQTLs in UKBEC and GTEx

We sought to determine if SNPs associated with schizophrenia and affective disorders also served as eQTLs in brain tissue. Study-SNPs identified in 11 GWAS [4–14] as well as SNPs in moderate to high LD [29] with these SNPs (see Methods) were included in this analysis (Fig 1). Combined, this yielded 106,397 analysis-SNPs across the 11 GWAS for eQTL interrogation. GWAS-independent and neurologically-disease free genotype and gene expression data were obtained from both UKBEC [25] and GTEx [26]. Of the 106,397 analysis-SNPs, 84,786 and 84,308 were present in UKBEC and GTEx, respectively. Expression data originated from four UKBEC and GTEx brain regions (cerebellum, frontal cortex, hippocampus and putamen).

To identify high confidence *cis*-eQTLs across regions in each study, we initially utilised an additive linear model with Matrix eQTL [31] in each of four regions within UKBEC and GTEx datasets, separately (S5 and S6 Figs). The number of detected *cis*-eQTLs using a stringent Bonferroni correction threshold varied considerably between studies and regions (S3 Table).

**Fig 2:**
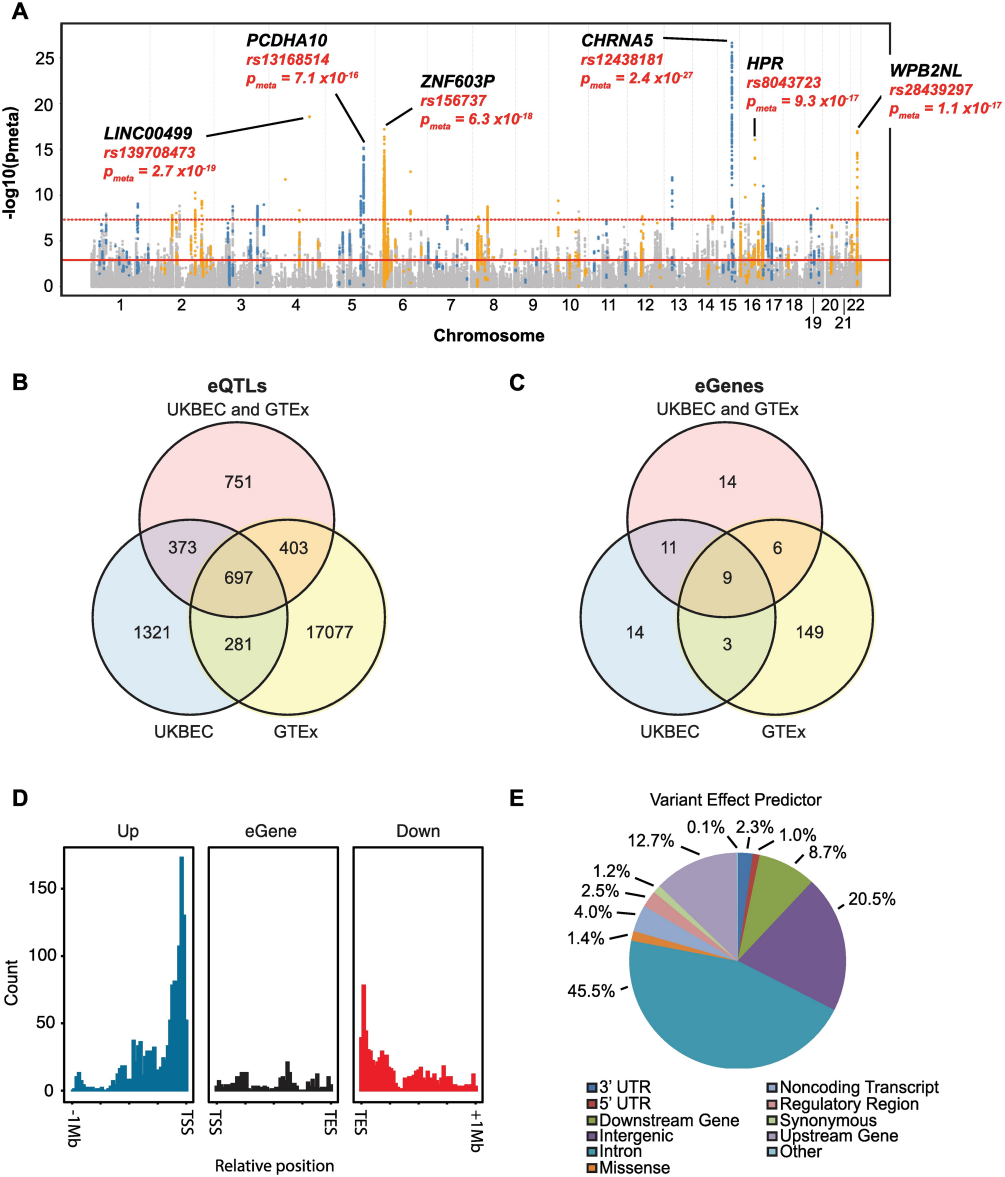
Manhattan plot and effect predictions of multi-region meta-analysis *cis*-eQTLs. *A*,–log10(p_meta_) are plotted for *cis*-eQTLs identified in a multi-region meta-analysis of eight brain regions from UKBEC and GTEX (Fig 1, analysis C). Top eGenes associated with these eQTLs are provided, along with the associated *cis-*eQTL and p_meta_. Solid red line denotes threshold at which FDR = 0.05. Dashed red line denotes threshold at which p-value < p_bonferroni_ (S4 Table). *B*, *C*, Overlap of *cis*-eQTLs and eGenes, respectively, between UKBEC+GTEx, UKBEC and GTEx with p_meta/ukbec/gtex_ < p_bonferroni_. *D*, Frequency of *cis*-eQTLs with respect to distance from associated eGene TSS (transcription start site) and TES (transcription end site). *E*, SNP effect prediction (from Ensembl Variant Effect Predictor) for 2,224 high-confidence *cis*-eQTLs.

We next leveraged these results across multiple regions within same datasets to increase discovery power and identify gene-SNP pairs that are *cis*-eQTLs across the four brain regions in each study [38, 39]. To do so, we first meta-analysed the single region *cis*-eQTLs across the four regions in UKBEC or GTEx separately (Fig 1, analyses A and B, S8 Fig, S5 and S6 Tables). Using a stringent Bonferroni correction threshold (S4 Table), 2,672 and 18,462 unique *cis*-eQTLs had p_ukbec/gtex_ < p_bonferroni_ in the UKBEC and GTEx data, respectively, associated with expression of 37 and 168 genes. Separately, we applied an alternative hierarchical bayesian MT-eQTL model that allows heterogeneity in both the distribution of eQTLs and their effect sizes across multiple tissues, and which additionally controls for correlated measurements of gene expression that can be apparent when sampling multiple tissues from the same donors [27, 40]. This model identified that 6,131 of 6,266 (97.8%) single-tissue eQTLs were highly likely in all four regions in UKBEC. In GTEx, 28,608 of 31,194 (91.7%) single-tissue eQTLs were highly likely in all four regions (S7a and S7b Fig). These were respectively associated with 74 and 282 genes. Finally, by intersecting both meta-analysis and MT-eQTL analysis, we identified 2,672 UKBEC and 18,458 GTEx *cis*-eQTLs present in all four tissues and with a p_ukbec/gtex_ < p_bonferroni_ (Fig 2B), associated with 37 and 167 eGenes, respectively (Fig 2C).

### High confidence eQTLs in multiple brain regions across studies

The aforementioned analyses were applied independently to both UKBEC and GTEx datasets. In order to generate a high-confidence list of eQTLs shared between datasets, as well as to discover additional ones, we next sought to determine if eQTLs identified in either UKBEC or GTEx data were significant when assessed across both studies. Following identical strategy to multi-region anaylsis on the separate study datasets, we first applied a meta-analysis of all *cis*-eQTLs present across eight regions (four UKBEC and four GTEx, Fig 1, analysis C). This identified 2,346 eQTLs with p_meta_ < p_bonferroni_ (S4 Table) associated with expression of 43 genes. Next, we applied the MT-eQTL model to these same eight regions (four UKBEC and four GTEx), and revealed that 18,340 of 20,347 (90.1%, S4 Table, S7c Fig) eQTLs were present in all tissues analysed and associated with 274 genes.

Intersection of both meta-analysis and MT-eQTL analysis identified 2,224 *cis*-eQTLs present in all four tissues across both studies that were associated with 40 eGenes (Fig 2A–2C, Table 1, S7 Table, S9 Fig). Of these, 1,070 *cis-*eQTLs, associated with 20 genes, were significant in the UKBEC multi-region intersection, whilst 1,100 *cis-*eQTLs, associated with 15 genes were also significant in the GTEx intersection (Fig 2B and 2C). Demonstrating reproducibility between the two studies, 697 of the 2,224 *cis*-eQTLs were also significant in UKBEC and GTEx (Fig 2B). Therefore, an additional 751 *cis*-eQTLs were detected when both UKBEC and GTEx datasets were leveraged in combination, thus demonstrating the benefit of a combined analysis. Indeed, this yielded discovery of 14 regulated genes not detected by either dataset alone (Fig 2C). Collectively we consider these 2,224 *cis*-eQTLs and 40 eGenes as the set of high confidence candidates to take forward. Importantly, a 10,000-fold permutation analysis validated these *cis*-eQTLs.

**Table 1:** Most significant multi-tissue *cis*-eQTL for each associated eGene.

eGene	Gene Function	eQTL	Alleles^†^	eQTL Location	β ± se	p_meta_	FDR	Associated Disease(s)
***CHRNA5***	Ligand-gated ion channel	rs12438181	G/A	Chr 15, *AGPHD1* intron	0.28 ± 0.03	2.44E-27	1.75E-21	SCZ
***LINC00499***	Non-coding RNA	rs139708473	G/A	Chr 4, *LINC00499* intron	-0.51 ± 0.06	2.72E-19	3.69E-15	MDD
***ZNF603P***	Non-coding RNA	rs156737	A/G	Chr 6, intergenic	-0.4 ± 0.05	6.66E-18	7.27E-14	SCZ
***WBP2NL***	Sperm-specific WW domain-binding protein	rs28439297	C/T	Chr 22, *NDUFA6-AS1* intron	-0.13 ± 0.02	1.11E-17	1.17E-13	SCZ
***HPR***	Hemoglobin binding	rs8043723	C/T	Chr 17, intergenic	-0.21 ± 0.03	9.26E-17	7.63E-13	MDD
***PCDHA10***	Calcium-dependent cell-adhesion	rs13168514	G/T	Chr 5, intergenic	-0.28 ± 0.03	7.08E-16	5.53E-12	SCZ, MDD
***ZSCAN31***	Transcription factor	rs276362	A/C	Chr 6, intergenic	-0.17 ± 0.02	4.98E-15	3.51E-11	SCZ
***HIST1H3E***	Nucleosome component	rs67575965	A/G	Chr 6, intergenic	-0.27 ± 0.04	1.27E-13	5.43E-10	SCZ
***BTN3A2***	Immunoglobin superfamily	rs3752417	G/C	Chr 6, *HIST1H3C* synonmous variant	0.15 ± 0.02	1.65E-13	6.61E-10	SCZ, CD
***C6orf3***	Non-coding RNA	rs6912446	T/A	Chr 6, *TRAF3IP2* intron	0.16 ± 0.02	2.68E-13	1.04E-09	SCZ
***SMIM2-AS1***	Non-coding RNA	rs4432167	G/C	Chr 13, intergenic	-0.21 ± 0.03	1.15E-12	3.77E-09	CD
***SRD5A3***	Steroid 5-alpha and polyprenol reductase families	rs2087319	C/A	Chr 4, intergenic	-0.15 ± 0.02	1.89E-12	5.63E-09	MDD
***SRR***	Catalyzes the synthesis of D-serine	rs4606747	T/C	Chr 17, *SRR* intron	-0.17 ± 0.02	1.00E-11	2.21E-08	SCZ
***AC068039*.*4***	Non-coding RNA	rs6738445	C/T	Chr 2, *DYNC1I2* intron	-0.47 ± 0.07	5.38E-11	9.02E-08	SCZ
***LINC01184***	Non-coding RNA	rs36694	A/C	Chr 5, intergenic	0.12 ± 0.02	1.30E-10	2.01E-07	BPD
***RRN3***	Transcription initiation	rs4985147	C/T	Chr 16, intron in RRN3 and PDXDC1	-0.1 ± 0.02	1.66E-10	2.49E-07	SCZ
***GAS8***	Cytoskeletal linker	rs4785709	G/A	Chr 16, *ZNF276* intron	0.08 ± 0.01	3.87E-10	4.99E-07	BPD, CD
***MASTL***	Serine/threonine kinase	rs7080612	G/T	Chr 10, *ANKRD26* intron	0.16 ± 0.03	4.07E-10	5.06E-07	SCZ
***TYW5***	tRNA hydroxylase	rs1704190	A/G	Chr 2, intergenic	0.1 ± 0.02	4.32E-10	5.18E-07	SCZ
***CD46***	Cofactor of complement factor I	rs1318653	T/C	Chr 1, intergenic	0.22 ± 0.04	8.63E-10	9.20E-07	SCZ
***RP11-275H4*.*1***	Non-coding RNA	rs1878874	A/T	Chr 3, *SOX2-OT* intron	-0.19 ± 0.03	1.07E-09	1.12E-06	SCZ
***RSRC1***	Pre-mRNA splicing	rs12491598	C/A	Chr 3, *AK097794* non-coding variant	0.11 ± 0.02	1.51E-09	1.48E-06	MDD
***LOC100130345***	Protein coding of unclear function	rs832190	T/C	Chr 3, *THOC7* intron	-0.1 ± 0.02	1.65E-09	1.55E-06	SCZ
***DDHD2***	Phospholipase enzyme	rs11779986	A/G	Chr 8, *BAG4* intron	-0.13 ± 0.02	1.75E-09	1.57E-06	SCZ, CD
***ZNF155***	Gene expression	rs1233454	G/A	Chr 19, *ZNF235* intron	0.11 ± 0.02	2.88E-09	2.43E-06	SCZ
***LRRC37A15P***	Non-coding RNA	rs223340	A/G	Chr 4, *UBE2D3* intron	-0.13 ± 0.02	4.40E-09	3.46E-06	SCZ
***HIST1H4H***	Nucleosome component	rs198855	T/A	Chr 6, intergenic	0.12 ± 0.02	5.85E-09	4.16E-06	SCZ
***ADAMTSL3***	Peptidase activity	rs4842841	G/A	Chr 15, *ADAMTSL3* intron	0.1 ± 0.02	6.37E-09	4.41E-06	SCZ
***LMAN2L***	Early secretory pathway	rs878919	T/C	Chr 2, *CNNM4* intron	-0.1 ± 0.02	9.00E-09	5.98E-06	BPD
***ALMS1P***	Non-coding RNA	rs11893881	T/A	Chr 2, *ALMS1* intron	-0.14 ± 0.02	1.52E-08	9.02E-06	SCZ
***MAU2***	Association of cohesion complex with chromatin during interphase	rs1009136	G/A	Chr 19, *MAU2* intron	0.06 ± 0.01	1.58E-08	9.34E-06	SCZ
***RP11-776H12*.*1***	Non-coding RNA	rs7525211	A/T	Chr 1, intergenic	0.12 ± 0.02	1.67E-08	9.76E-06	BPD
***TMEM81***	Protein coding of unclear function	rs12143085	A/G	Chr 1, *RBBP5* intron	0.16 ± 0.03	1.87E-08	1.07E-05	SCZ
***RP11-3N2*.*13***	Non-coding RNA	rs10257979	A/C	Chr 7, *ZNF727* intron	0.18 ± 0.03	1.92E-08	1.10E-05	SCZ
***SPATA7***	Photoreceptor cell maintenance	rs11623942	G/C	Chr 14, intergenic	0.12 ± 0.02	1.94E-08	1.11E-05	SCZ
***ATP5G2***	Mitochondrial ATP synthase subunit	rs10876460	A/G	Chr 12, *PCBP2* intron	0.09 ± 0.02	2.04E-08	1.15E-05	SCZ
***AF131216*.*5***	Non-coding RNA	rs11249996	A/C	Chr 8, *MSRA* intron	-0.07 ± 0.01	2.24E-08	1.24E-05	SCZ
***MNT***	Transcriptional repressor	rs413016	C/T	Chr 17, *TSR1* intron	-0.08 ± 0.02	2.80E-08	1.46E-05	SCZ
***GSTO2***	Glutathione-dependent thiol transferase activity	rs4925	C/A	Chr 10, *GSTO1* missense variant	0.21 ± 0.04	3.35E-08	1.67E-05	SCZ
***TOM1L2***	Vesicular trafficking	rs11652881	A/G	Chr 17, *RAI1* intron	0.09 ± 0.02	4.19E-08	1.95E-05	SCZ

^†^First allele listed is the reference allele for effect size. For example, relative to *G*, *A* has a β of 0.28 ± 0.03 EU per allele with respect to expression levels of *CHRNA5*.

### Disease-associated eQTL characteristics

Of the 2,224 cis-eQTLs, 58% (1,292) were located with 1MB upstream of the eGene TSS (transcription start site), 11% were located within the eGene itself while the remaining 31% were downstream of the TES (transcription end site). Positional analysis relative to the associated eGenes showed that *cis*-eQTLs were most enriched within 250kb of the TSS and TES, respectively (Fig 2D). With respect to eQTL variant positions, 45.5% were localised to introns (Fig 2E), as classified by Ensembl Variant Effect Predictor. This high proportion of introns likely includes overlap with introns of neighbouring transcripts. Both observations are consistent with other studies [33, 34]. No clear gene ontology was associated with the eGenes.

**Fig 3:**
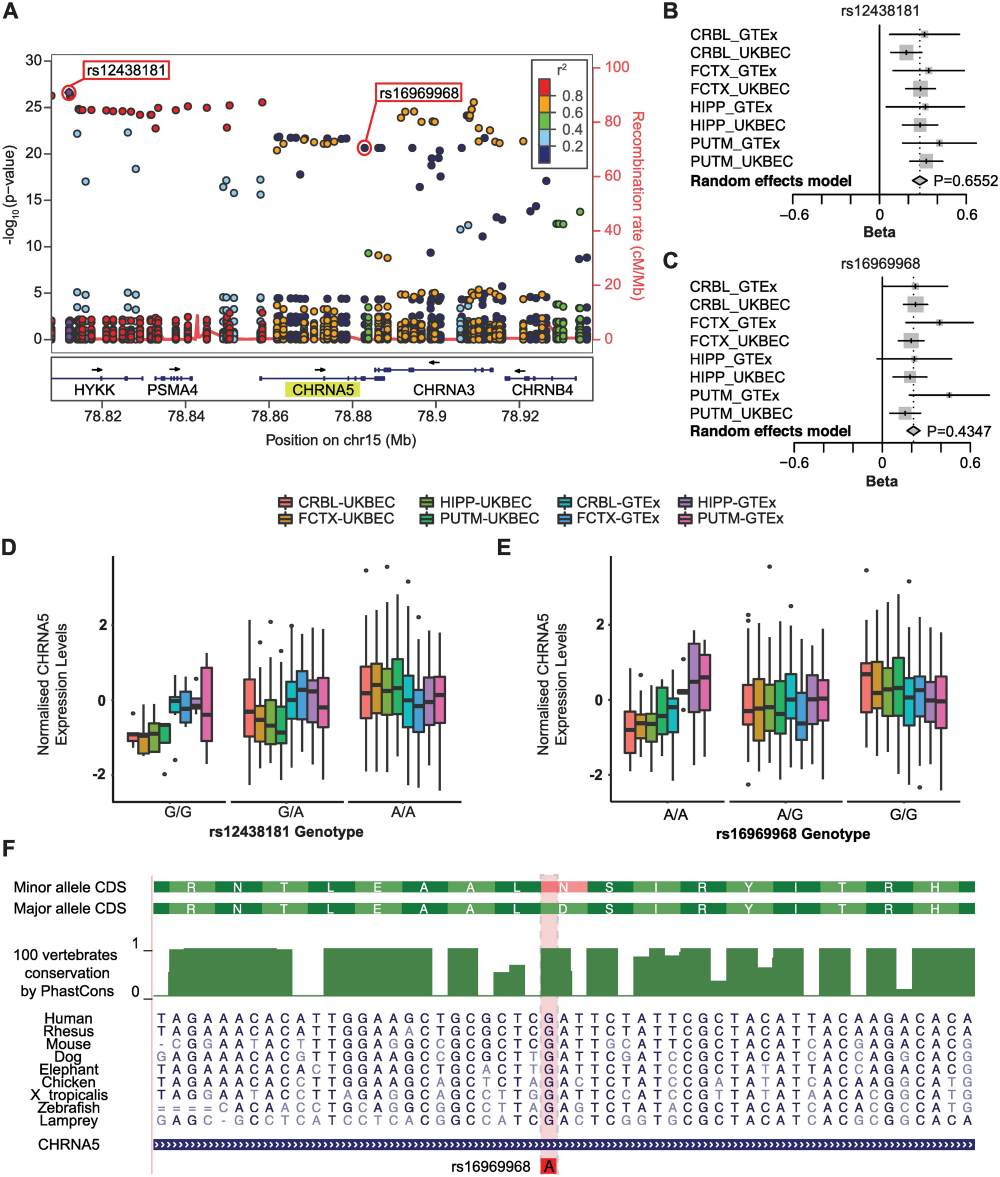
Mapping and gene expression effects of *CHRNA5* associated eQTLs. *A*, LocusZoom plot for the most significant *cis*-eQTL (rs12438181) identified in the multi-region meta-analysis of UKBEC and GTEx (Fig 1, analysis C; Table 1). This SNP is *cis* to *CHRNA5*, denoted in yellow highlight. rs16969968, the *cis*-eQTL leading to a missense mutation in CHRNA5, is also identified. Other SNPs that are within the study and *cis* to *CHRNA5* are plotted for their p_meta_ (left y-axis) and LD-value (r^2^ denoted by colour of circle, calculated as relative to rs12438181). Recombination rate of genomic region is plotted in red. *B*, *C*, Summarized forest plots of random effects meta-analysis for rs12438181 and rs16969968, respectively. *D*, *E*, Boxplot of gene expression levels (normalised separately for UKBEC and GTEx per region) by genotype of the rs12438181 and rs16969968 eQTLs, respectively. Vertical lines for each plot capture data between -1.5 x interquartile rage and 1.5 x interquartile range, with outliers depicted as black points. The number of individuals with a particular genotype ordered by the illustrated study-regions for rs12438181 were GG: 5/5/5/5/6/4/4/6, GA: 41/40/33/34/29/23/21/21, AA: 84/81/74/78/50/45/41/41. The number of individuals with a particular genotype per study-region for rs16969968 were AA: 19/17/10/14/10/5/6/4, AG: 61/57/56/55/29/25/22/24, GG: 50/52/46/48/47/43/38/41. *F*, Amino acid conservation plot for CHRNA5. Missense mutation resulting from the eQTL rs16969968 is highlighted. *CRBL*, cerebellum; *FCTX*, frontal cortex; *HIPP*, hippocampus; *PUTM*, putamen.

Mean and median of the (absolute value of) effect sizes of candidate eQTLs were 0.26 and 0.25 EU per allele, respectively (S10 Fig). eQTLs positioned within 250kb of the TSS and TES, respectively, had the greatest absolute effect size (S11 Fig), similar to what was seen with respect to relative TSS position and variant frequency (Fig 2D). The largest effect size was observed with rs9461434 (-0.56 ± 0.09 EU per allele for *AA* relative to *CC*), associated with the expression of the pseudogene *ZNF603P* (45kb upstream of the TSS, p_meta_ = 3.7 x 10^−9^, FDR = 2.9 x 10^−6^, S12 Fig). The most significant *cis-*eQTL was rs12438181 on chromosome 15q25.1, 46kb upstream and correlated with the expression of *CHRNA5* (p_meta_ = 2.4 x 10^−27^, FDR = 1.8 x 10^−21^), which encodes the α5 subunit of the nicotinic cholinergic receptor. This eQTL had an effect size of 0.28 ± 0.03 EU per allele for *AA* relative to *GG* (Fig 3A, 3B and 3D, S9 Fig).

Interestingly, 28 *cis*-eQTLs (S8 Table) were classified as leading to missense mutations. Three eQTLs resulted in missense mutations within genes whose expression they were then also additionally associated. The most significant of these was rs16969968 (p_meta_ = 2.3 x 10^−21^), which is located within exon 5 of *CHRNA5*, a highly conserved region (Fig 3C, 3E and 3F, S9 Fig). The minor allele *A* encodes for an amino acid change to asparagine from aspartic acid (major allele *G*) at position 398, which may affect receptor function (see Discussion). The effect size of *GG*, relative to *AA*, is 0.21 ± 0.02 EU per allele, thus implying that both expression changes and receptor activity could be contributing to the effect of this variant.

### Replication of eQTLs across other brain tissue studies

To assess replicability with other eQTL studies using brain tissue, we evaluated our findings relative to similar studies. Previously, 27 brain eQTLs were identified in a meta-analysis of GWAS SNPs associated with five neuropsychiatric disorders (including three studied here [14]) using cortical expression data from five separate studies [41]. We identified one of these overlapping with our study: rs4523957 associated with expression of *SRR* on chromosome 17p13.3. Though this was not the most significant eQTL for this gene, thus suggesting that the lead eQTL and the GWAS signals might be different (see below), the association still surpassed the Bonferroni threshold with p_meta_ = 8.1 x 10^−11^, FDR = 1.3 x 10^−7^. *SRR* encodes for an enzyme that converts L- to D-serine, which has be found to be lower in the CSF of patients with schizophrenia [42], supporting the role of glutamatergic neurotransmission in the biology of schizophrenia and affective disorders [43, 44]. Indeed, it is also a candidate drug target for schizophrenia, again highlighting the potential for eQTL studies to identify pathobiology that might be targeted pharmacologically. *ZSCAN31*, also known as *ZNF323*, is another *cis*-eQTL associated gene that has been previously identified as significantly associated with schizophrenia, bipolar disorder and psychosis in both a GWAS [45] and an eQTL study using 193 human prefrontal cortex samples [46]. In our study, multiple *cis*-eQTLs associated with this gene were significant (p_meta_ = 5.0 x 10^−15^ for the top eQTL). Likewise, two genes that were prioritized as putatively causal from integrative analysis of PGC-SCZ2 GWAS with both whole blood and UKBEC data averaged across 10 brain regions [47], *SNX19* and *NMRAL1*, were associated with *cis*-eQTLs with some evidence of significance, p_meta_ = 1.6 x 10^−4^ and 6.5 x 10^−6^, respectively (both genes had probable eQTLs in all eight brain regions analysed across UKBEC and GTEx).

Next, we sought to test replication of our high-confidence candidate eQTLs in a disease-associated eQTL study. Here, the CommonMind Consortium (CMC) study [34] recently analysed gene expression of dorsolateral prefrontal cortex in over 500 schizophrenia cases and controls. In comparing the two analyses, of the 2,224 eQTLs surpassing the p_meta_ Bonferroni threshold, 1,969 (88.5%) had a FDR_CMC_ < 0.05, accounting for 30 out of 40 eGenes identified in our study (S13A Fig). Importantly, 7,555 of the 10,286 eQTLs with FDR_meta_ < 0.05 were also significant in the CMC study, suggesting strong overlap between the two analyses despite our use of healthy brain tissue alone (π_1_ = 0.73, S9 Table). The most significant overlapping eQTL was rs139708473 associated with the expression of *LINC00499*, a brain and testis expressed non-coding RNA on chromosome 4q28.3 with unclear function (p_meta_ = 2.7 x 10^−19^, FDR = 3.7 x 10^−15^, effect size = -0.51 ± 0.06 EU per *AA*, compared to *GG*, S13B Fig).

### Overlap with eQTLs from peripheral tissues

A key consideration in eQTL analyses is the source of tissue used for gene expression data. While identification of eQTLs is increased by studying associations in multiple tissues [48, 49], other studies have found that analyses in disease-related tissues are enriched for disease-associated eQTLs [50, 51]. Recent availability of datasets detailing gene expression in various regions of human brain have now allowed for eQTL analyses in nervous tissue [25, 26, 52]. Previous eQTL analyses for schizophrenia and affective disorders have relied on transcriptome data collected from more readily available biological specimens such as whole blood [53–55] or more disease-related specimens such as single brain regions [55]. An important and clinically relevant question is whether disease-related eQTLs can be detected in samples that are more accessible and related from living patients. If so, this may facilitate larger study designs in the future where high quality biological material is more readily selected.

**Fig 4:**
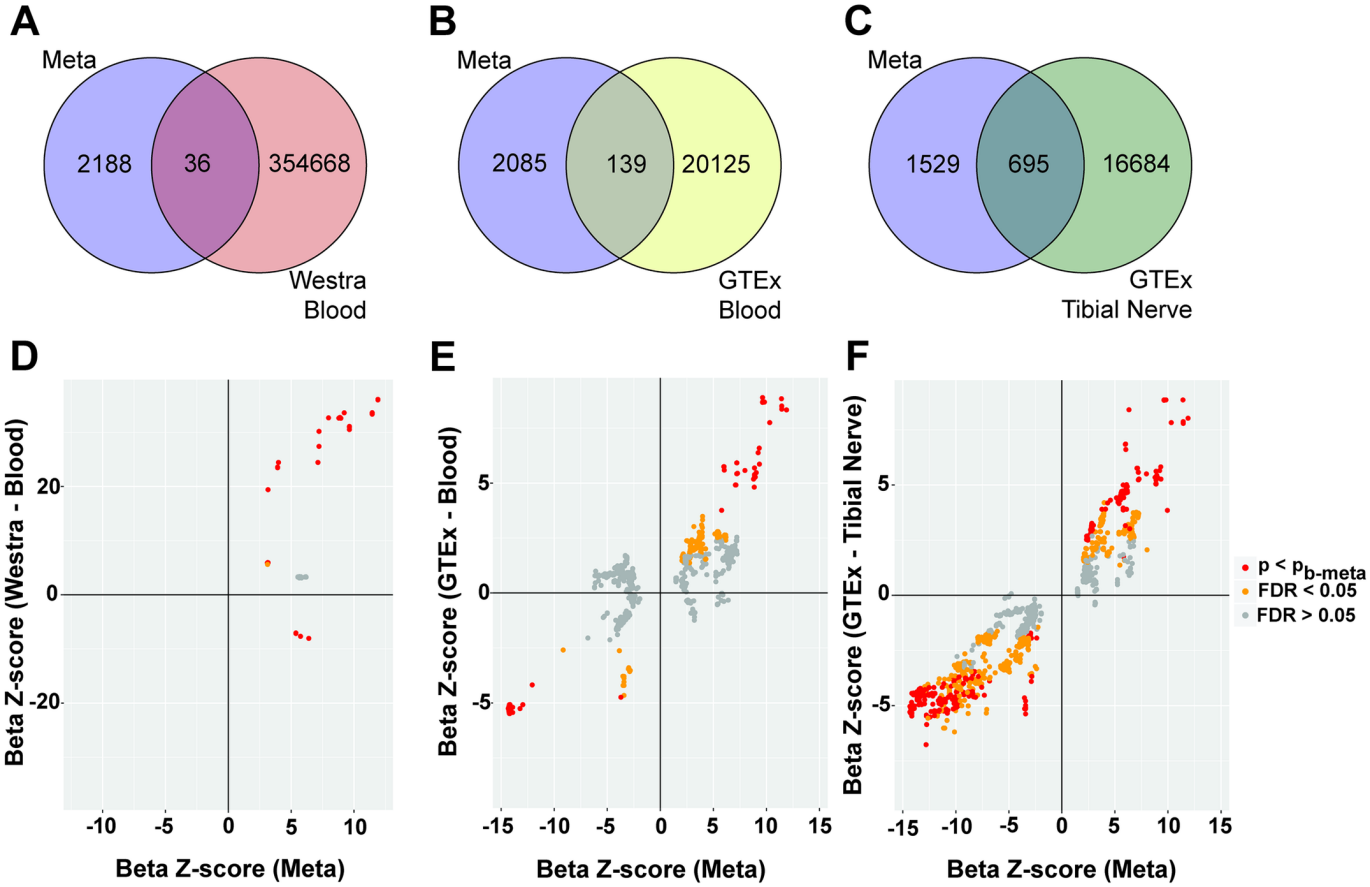
Overlap of multi-region meta-analysis *cis*-eQTLs with those from non-brain tissues. *A-C*, Venn diagrams for number of distinct and overlapping *cis*-eQTLs between multi-region meta-analysis (Meta, Fig 1, analysis C) and eQTLs Blood eQTL Browser (Westra Blood), GTEx whole blood tissue (GTEx Blood) and GTEx Tibial Nerve, respectively. *D-F*, Scatterplot of beta z-scores of Meta and Westra Blood, GTEx Blood and GTEx Tibial Nerve, respectively. Points are coloured by the *cis*-eQTL p- and FDR-values from the y-axis analyses. *p*_*b-meta*_ is the Bonferroni correction threshold for p_meta_.

To test whether our significant *cis*-eQTLs detected in brain regions can be identified in more clinically-accessible tissue, we assessed the associations of the 2,224 *cis*-eQTLs in whole blood tissue. Of these, only 50 were detected in the largest meta-analysis of peripheral whole blood to date (Westra Blood, n = 5,311) [32], with 36 (1.6%) reaching significance in the Westra Blood samples (Fig 4A and 4D, S14D and S14G Fig). These eQTLs were associated with four genes (*BTN3A2*, *HIST1H4H*, *CHRNA5* and *GSTO2*) (S15B Fig). Overall, replication of our data in the Westra Blood samples was very low (π_1_ = 0.05). Of note, eQTLs associated with *CHRNA5* expression had a minimum p_blood-Westra_ > 7 x 10^−4^.

Similar results were observed within the GTEx database, where only 1,146 of the 2,224 *cis-*eQTLs were detected in GTEx-whole blood. However, only 139 (6.3%) reached the stringent Bonferroni correction threshold for GTEx-whole blood (Fig 4B and 4E, S14E and S14H Fig, π_1_ = 0.32). Looking in more detail, of the 40 genes associated with brain *cis*-eQTLs from the multi-region meta-analysis, thirty had detectable expression in the GTEx whole blood samples. However, only *BTN3A2*, *SRD5A3* and *DYNC1I2* were associated with blood *cis*-eQTLs having p_blood-GTEx_ < p_bonferroni_ (S14B and S15A Figs). The most significant brain *cis*-eQTL in whole blood was rs72841536 (chromosome 6p22.2), which correlated with *BTN3A2* expression (p_blood-GTEx_ = 2.7 x 10^−46^ and FDR = 5.1 x 10^−43^). Of note, the direction of the effect was the same as that seen in the brain eQTL; the effect size was 0.86 ± 0.05 EU per allele in blood compared to an effect size of 0.44 ± 0.08 EU per allele in brain (S15C and S15D Fig). Contrastingly, the top brain *cis*-eQTL, rs12438181, associated with *CHRNA5* expression, only had a p_blood-GTEx_ = 0.05 and FDR = 0.40 (effect direction was the same). Thus, whilst brain-related *cis*-eQTLs can be detected in whole blood in principle, nearly 50% were not detected and over 90% did not reach significance in whole blood analyses.

Although it has been demonstrated that gene expression profiles of the central nervous system (CNS) are different than those of the peripheral nervous system (PNS) [56, 57], data suggest that the transcriptomes of the CNS and PNS are more similar than that between CNS and blood [26]. These findings, along with increased tissue accessibility of the PNS over the CNS, motivated us to ascertain whether significant *cis*-eQTLs captured in the multi-region meta-analysis were also significant in eQTL analysis of tibial nerve tissue, a component of the PNS. Of the 2,224 *cis*-eQTLs, 2,220 were detected in GTEx tibial nerve tissue and more importantly, 695 (31.2%) reached the Bonferroni correction threshold for GTEx tibial nerve (Fig 4C and 4F, S14F and S14I Fig, S10 Table, π_1_ = 0.66), five-fold more than that detected in whole blood. Moreover, 38 of 40 significantly associated eGenes had detectable expression in the GTEx tibial nerve samples (*LINC00499* and *AF131216*.*5* were not detected in tibial nerve samples). Importantly, seven genes (*ZNF603P*, *DDHD2*, *ALMS1P*, *RSRC1*, *AC068039*.*4*, *CD46*, *LMAN2L*, S10 Table) were associated with overlapping significant eQTLs (p_meta_ and p_nerve-GTEx_ both < p_bonferroni_, S16 Fig). This suggests that while the tibial nerve does not fully capture the eQTLs identified in brain, it may be a better proxy than whole blood. Taken together, these findings strongly suggest that eQTL analyses should either be performed in disease-relevant tissue wherever possible or that more extensive studies targeting minimally invasive tissues, such as those from the PNS, are necessary to identify suitable brain correlates.

## Discussion

In this multi-region meta-analysis, we report the presence of eQTLs in brain tissue for SNPs that are associated with a risk of developing schizophrenia and affective disorders. Even with the requirement of a stringent Bonferroni correction p-value threshold and being an eQTL in all tissues assessed, we identified 2,224 *cis*-eQTLs that were correlated with expression of 40 genes. These associations held across four brain regions from two independent studies (UKBEC and GTEx) of neurologically-normal individuals as well as through permutation analyses of 10,000 iterations, supporting the robustness of these findings.

Of the 2,224 *cis*-eQTLs detected, nearly two-thirds were located within introns or intergenic regions, consistent with previous studies [55, 58]. Meanwhile, 1.4% were classified as causing missense mutations in the proteins encoded from genes harbouring the eQTL. The most significant eQTL that results in a missense mutation within the gene whose expression it is associated with, rs16969968, leads to an amino acid change at position 398 in *CHRNA5*, a subunit in nicotinic acetylcholine receptors. Interestingly, functional *in vitro* studies have demonstrated that receptors containing this missense mutation are less responsive to a nicotinic agonist than ones with the more common variant [59, 60]. This leads to reduced cell-depolarization and cholinergic signalling and is consistent with the reported hypofunction of cholinergic signalling in schizophrenia [61]. Moreover, pharmaceutical modulation of cholinergic signalling may improve outcomes for schizophrenia [62].

There is modest evidence that this and other eQTLs affecting *CHRNA5* expression are associated with schizophrenia and affective disorders [6, 63, 64]. However, rs16969968 has also been shown to be associated with increased tobacco use [59, 65] and incidence of lung cancer [66, 67]. Therefore, given the disproportionate percentage of individuals with mental illness that smoke [68], further studies are needed to ensure that these eQTLs are not associated with a confounding behaviour seen in such individuals. Nonetheless, these *cis-*eQTLs for *CHRNA5* demonstrate how genetic analyses can identify variants that may increase disease-risk while concurrently being potential therapeutic targets.

Another clinically interesting eQTL is that of rs12491598, associated with the expression of *RSRC1* on chromosome 3 (S17 Fig). While this SNP was initially identified in a GWAS of MDD [13], the eGene from our eQTL-analysis has been associated with schizophrenia in a combined case-control imaging genetics study utilising left dorsal lateral prefrontal cortex activation as an intermediate phenotype [69, 70]. *RSRC1* is involved in pre-mRNA splicing [71] as well as a marker of subventricular zone progenitor cells within the foetal and postnatal forebrain [72], supporting the hypothesis of a developmental aetiology for schizophrenia. However, SNPs *cis* to this gene were not significantly associated with schizophrenia [6], with a minimum p-value of 1.4 x 10^−3^. Moreover, *RSRC1* has also been associated with the extreme ranges of height [73]. These findings necessitate further investigation of the role of this gene in health and disease.

More generally, comparative analysis found that our approach replicated several *cis*-eQTLs found in other studies. This includes the well-powered report of the CommonMind Consortium [34], which utilised both healthy and diseased brain samples to discover eQTLs in the dorsolateral prefrontal cortex. Indeed, 1,969 (88.5%) had a FDR_CMC_ < 0.05, accounting for 30 out of 40 eGenes identified in our study (π_1_ = 0.73). This supports our approach of leveraging information across eQTL studies to discover new regulatory elements that aids interpretation of GWAS results. Indeed, these findings also support the suitability of non-disease state tissue to investigate disease pathobiology, as has been demonstrated with the use of normal prostate tissue to study prostate cancer [74].

Despite the highlighted overlaps with previous studies, several reported associations were not significant here. *CACNA1C* and *ZNF804A* are two genes that have been implicated in schizophrenia and affective disorders through multiple studies, including GWAS and case/control brain expression analyses [75–79]. While both UKBEC and GTEx data contain expression data regarding these genes and the set of analysis-SNPs contain variants that are in *cis*, we did not find any significant eQTLs in our meta-analysis of UKBEC and GTEx across the four overlapping brain regions. For *CACNA1C* (minimum p_meta_ = 1.3 x 10^−3^), the effect size estimates of the *cis-*eQTLs had different signs across the various tissues and studies (effect size of 0.06 ± 0.02 EU per allele, S11 Table). Similarly, for *ZNF804A*, the effect size estimates also demonstrated different signs across the tissues and studies (p_meta_ = 3.1 x 10^−1^, effect size of -0.02 ± 0.02 EU per allele for the most significant meta-eQTL, S12 Table). This suggests that some disease-implicated *cis*-eQTLs may have varying effects in different brain regions, warranting further investigation. Case inclusion may also be a confounding issue when comparing to some studies. Suggestive of this, overlap of our eQTLs was less apparent in the longitudinal ROSMAP study [33] that had reported eQTLs from the dorsolateral prefrontal cortex (π_1_ = 0.40). We note that despite the 500 study individuals that were healthy at the time of enrolment in ROSMAP, over half developed Alzheimer’s disease by the time brain tissue was donated for analysis. Further investigation will therefore be required to determine if sample size and/or confounding disease genetics could be the reason for limited intersection of these two eQTL studies, especially given the intense research into the proportion of disease- and tissue-specific eQTLs [34, 80, 81].

It is important to determine which specific isoforms are the target of the *cis-*eQTLs identified in this study [34], and which are the causal variants associated with GWAS signal. Fine-mapping tools such as SHERLOCK [82], RTC [83], Coloc [36] and eCAVIAR [84] have been developed to achieve the latter, and have so far been used to prioritize certain genes in schizophrenia and affective disorders [34, 47, 85]. A Coloc analysis of our high-confidence eQTLs with the PGC-SCZ2 schizophrenia GWAS supported colocalisation between the GWAS and lead eQTL signals for seven of the thirty-three eGenes (S13 Table). This ratio is comparable to other studies which demonstrate that less than a fifth of GWAS loci for schizophrenia have had genes prioritized in this way. However, it is also important to appreciate that current estimates suggest 5–25% of credible intervals identified with such tools may not actually contain the causal variants [86]. Moreover, many *cis-*eQTLs are driven by multiple independent SNPs [58, 87, 88]. As Coloc assumes at most one causal variant per region [36], it may be the case that many of the signals that we have captured as high-confidence eQTLs are secondary or tertiary. This remains an area we are further investigating computationally. Further, this strongly encourages follow up experimental validation to help elucidate causal SNPs and genes [34]. Meanwhile, emerging research showing integrated epigenome and transcriptome QTL analysis can complement fine-mapping approaches for variant prioritization is an exciting new avenue that merits further exploration [33, 89].

With reference to future QTL studies, progress in understanding the biology governing schizophrenia and affective disorders has been hampered by difficulty in accessing disease-relevant tissue. Therefore, peripheral blood, as it is more accessible, has previously been used as a proxy for studying eQTLs of complex diseases [21, 32, 47]. However, it is unclear as to how robust findings from blood samples are with respect to studying diseases outside of the hematopoietic system. By comparing *cis*-eQTL results from brain and blood, we find that a minority of the brain-eQTLs were detected in blood and their significance (as marked by p-value) was greatly diminished. Substantially more overlap (over >99% of brain-eQTLs) was demonstrated in GTEx tibial nerve samples, with 31.2% having significant association in both analyses. The extent to which this overlap is driven by power versus tissue specificity is unclear. Therefore, studying eQTLs in disease-related tissues (i.e. central or peripheral nervous tissue in this case) is warranted and may prioritize eQTLs for further validation and mechanistic studies. Indeed, this has been demonstrated through the identification of eQTLs associated with prostate cancer with the use of non-diseased prostate tissue [74].

In summary, we have identified robust *cis-*eQTLs associated with schizophrenia and affective disorders in human brain tissue. Of the eQTL-associated genes, many have been implicated previously, such as *CHRNA5* and *RSRC1*, while others are novel associations (i.e. *ZNF603P*) that now merit further analyses. We also demonstrate that eQTL analysis in disease-related tissues allows for prioritization of associations for follow-up studies in diseased-tissue. These results provide insight into putative mechanisms related to development of schizophrenia and affective disorders, thereby identifying potentially new therapeutic targets.

## Supporting information

S1 Fig Principal component analysis of cerebellum data from UKBEC-GTEx meta-analysis. First ten principal components (PC) are shown. Red points, GTEx; black points; UKBEC.
(TIF)

S2 Fig Principal component analysis of frontal cortex data from UKBEC-GTEx meta-analysis. First ten principal components (PC) are shown. Red points, GTEx; black points; UKBEC.
(TIF)

S3 Fig Principal component analysis of hippocampus data from UKBEC-GTEx meta-analysis. First ten principal components (PC) are shown. Red points, GTEx; black points; UKBEC.
(TIF)

S4 Fig Principal component analysis of putamen data from UKBEC-GTEx meta-analysis. First ten principal components (PC) are shown. Red points, GTEx; black points; UKBEC.
(TIF)

S5 Fig QQ plot for UKBEC brain regions. Theoretical (x-axis) versus observed (y-axis) Matrix eQTL calculated p-values (-log10) for each analysed region in UKBEC data. Red points, *cis*-eQTLs; blue points, *trans-*eQTLs; grey line represents null line. *CRBL*, cerebellum; *FCTX*, frontal cortex; *HIPP*, hippocampus; *PUTM*, putamen.
(TIF)

S6 Fig QQ plot for GTEx brain regions. Theoretical (x-axis) versus observed (y-axis) Matrix eQTL calculated p-values (-log10) for each analysed region in GTEx data. Red points, *cis*-eQTLs; blue points, *trans-*eQTLs; grey line represents null line. See S5 Fig for abbreviations.
(TIF)

S7 Fig *cis-*eQTL tissue distribution from MT-eQTL analyses. *cis*-eQTLs were analysed using the MT-eQTL model to identify the distribution amongst the tissues. Cerebellum, frontal cortex, hippocampus and putamen were assessed for each UKBEC (*A*) and GTEx (*B*). All eight regions were assessed for the UKBEC + GTEx analysis (*C*). A majority of the *cis*-eQTLs were present in all tissues assessed (four for UKBEC and GTEx and eight for UKBEC + GTEx).
(TIF)

S8 Fig Manhattan plot of *cis*-eQTLs meta-analysis of UKBEC and GTEx brain regions. *cis-*eQTLs from meta-analysis of four UKBEC (*A*) and GTEx (*B)* brain regions (cerebellum, frontal cortex, hippocampus and putamen), plotted for p_ukbec/gtex_ (-log10). Colored point (blue and yellow) indicate eQTLs that are multi-region eQTLs in the four brain regions. Grey points indicate eQTLs that are not multi-region eQTLs in all four regions. Dashed red line indicates level where p-value < p_bonferroni-ukbec/gtex_; solid red line indicates p-value at which FDR = 0.05. Note different scales for y-axes.
(TIF)

S9 Fig Forest plots of significant *cis*-eQTLs from meta-analysis of overlapping regions in UKBEC and GTEx. *A-MO*, Forest plots, by study, of 41 *cis*-eQTLs through multi-region meta-analyses. Each plot is denoted by the eQTL and corresponding eGene (i.e. rs139708473 –*LINC00499* for *A*). Note that summarised forest plots for *cis*-eQTLs rs12438181 –*CHRNA5* (*MN*) and rs16969968 –*CHRNA5* (*MO*) are shown in Fig 3B and 3C. *C*, cerebellum; *F*, frontal cortex; *H*, hippocampus; *P*, putamen.
(PDF)

S10 Fig Distribution of *cis*-eQTL effect sizes. A, Absolute values of effect sizes (beta, standardised expression units per allele) plotted against p_meta_ (-log10) for *cis*-eQTLs detected in multi-region meta-analyses. Red dashed line indicates p_bonferroni-meta_. B, Density plot of (absolute value of) effect sizes, regardless of p_meta_ values. Inset depicts density plot for eQTLs with p_meta_ < p_bonferroni-meta_. C, D, *cis*-eQTLs plotted for minor allele frequencies (calculated per study-region) and effect sizes per region for UKBEC and GTEx, respectively. Effect sizes are based on eQTL analysis per region and study. Red colored points represent significant *cis*-eQTLs from multi-region meta-analyses. CRBL, cerebellum; FCTX, frontal cortex; HIPP, hippocampus; PUTM, putamen.
(TIF)

S11 Fig Distribution of effect sizes relative to TSS. Scatter plot of absolute value of effect sizes (abs(Effect)) for the high-confidence eQTLs from the multi-region meta-analysis. Point are plotted with respect to the TSS (transcription start site) and TES (transcription end site) of the associated eGene.
(TIF)

S12 Fig rs9461434 eQTL boxplot for *ZNF603P* expression. Boxplot of *ZNF603P* expression levels (normalised separately for UKBEC and GTEx per region) by genotype of the rs9467434 eQTL. This eQTL had the largest magnitude effect size, -0.56 ± 0.09 EU per allele for *AA* relative to *CC*, in the multi-region meta-analysis of *cis*-eQTLs. Vertical lines for each plot captures data between -1.5 x interquartile rage and 1.5 x interquartile range, with outliers depicted as black points.
(TIF)

S13 Fig Overlap with CMC schizophrenia case-control study. *A*, CMC *cis*-eQTLs with FDR_CMC_ < 0.05 plotted for significance (-log10(p_meta_)) from multi-region meta-analysis significant eQTLs (Fig 1C). Grey points indicate MT-eQTL meta-analysis eQTLs with FDR_meta_ < 0.05 and present in all regions studied. Colored eQTLs, by eGene, represent those that are associated with eGenes from the high-confidence list (see main text). Red line indicates p_bonferroni_ threshold for the UKBEC+GTEx meta-analysis. *B*, Boxplot of *LINC00499* expression levels (normalised separately for UKBEC and GTEx per region) by genotype of the rs139708473 eQTL. This eQTL had the largest magnitude effect size, -0.51 ± 0.06 EU per allele for *AA* relative to *GG*, of the multi-region meta-analysis of *cis*-eQTLs that overlapped with CMC. Vertical lines for each plot captures data between -1.5 x interquartile rage and 1.5 x interquartile range, with outliers depicted as black points.
(TIF)

S14 Fig Overlap of eQTLs and eGenes between Meta-analysis and non-brain tissues. *A-C*, Venn diagram of eGenes associated with significant eQTLs (see Fig 4A–4C) for eQTLs from the Blood eQTL Browser (Westra Blood), GTEx whole blood and GTEx tibial nerve, respectively. *D-F*, Venn diagram of significant eQTLs from multi-region meta-analyses (Meta) irrespective of significance in eQTLs from Westra Blood, GTEx whole blood and GTEx tibial nerve, respectively. *G-I*, Number of eGenes associated with eQTLs from *D-F*.
(TIF)

S15 Fig P-value distribution of brain and blood *cis-*eQTLs. p-value plot of *cis-*eQTLs brain (p_meta_) versus *A*, GTEx sample of whole blood (p_blood-GTEx_) or *B*, meta-analysis of whole blood (p_blood-Westra_). eQTLs are coloured by associated eGene. Dashed grey lines indicate p_bonferroni-blood_ (horizontal line) and p_bonferroni-meta_ (vertical line). eQTL plots for rs72841536 for *BTN3A2* expression in GTEx whole blood samples (*C*) and in overlapping brain regions in UKBEC and GTEx samples (*D*). This eQTL was the most significant blood eQTL (in GTEx whole blood samples) that overlapped with *cis*-eQTLs identified through multi-region meta-analyses.
(TIF)

S16 Fig P-value and beta distributions of tibial *cis-*eQTLs. *A*, p-value plot of *cis-*eQTLs brain (p_meta_) versus GTEx tibial nerve samples (p_nerve-GTEx_). eQTLs are coloured by associated eGene. Dashed grey lines indicate p_bonferroni-meta_ (x-axis) and p_bonferroni-tibial_ (y-axis). *B*, Absolute values of effect sizes (beta, standardised expression units per allele) plotted against p_nerve-GTEx_ (-log10) for *cis*-eQTLs detected in GTEx tibial nerve samples. Red points indicate those *cis*-eQTLs significant in multi-region meta-analysis. Black dashed line indicates p_bonferroni-tibial_.
(TIF)

S17 Fig Mapping and gene expression effects of *RSRC1* associated eQTLs. *A*, LocusZoom plot for the *cis*-eQTL rs12491598, identified in multi-region meta-analyses (Table 1). This SNP is *cis* to *RSRC1*, denoted in yellow highlight. Other SNPs that are within the study and *cis* to *RSRC1* are plotted for their p_meta_ (left y-axis) and LD-value (r^2^ denoted by colour of circle, calculated as relative to rs12491598). Recombination rate of genomic region is plotted in red. *B*, Boxplot of gene expression levels (normalised separately for UKBEC and GTEx per region) by genotype of the rs12491598 eQTL. Vertical lines for each plot capture data between -1.5 x interquartile rage and 1.5 x interquartile range, with outliers depicted as black points. The number of individuals with a particular genotype per study-region is denoted below in italics. *CRBL*, cerebellum; *FCTX*, frontal cortex; *HIPP*, hippocampus; *PUTM*, putamen.
(TIF)

S1 Table GWAS datasets and number of SNPs. (XLSX)

S2 Table Sample size by brain region for UKBEC and GTEx. (XLSX)

S3 Table Summary of *cis*-eQTL analyses from single-region analyses. (XLSX)

S4 Table Summary of *cis*-eQTL analyses from multi-region analyses. (XLSX)

S5 Table Most significant *cis*-eQTL for each associated eGene from multi-region analysis in UKBEC. (XLSX)

S6 Table Most significant *cis*-eQTL for each associated eGene from multi-region analysis in GTEx. (XLSX)

S7 Table *cis*-eQTLs significant in multi-region meta-analysis of four overlapping regions between UKBEC and GTEx (Fig 1, analysis C). (XLSX)

S8 Table List of significant multi-region *cis*-eQTLs resulting in amino acid change. (XLSX)

S9 Table *cis*-eQTLs with FDR_meta_ < 0.05 in multi-region meta-analysis that are also significant in CommonMind Consortium schizophrenia case-control eQTLs, list by top eQTL per eGene. (XLSX)

S10 Table Overlap of *cis*-eQTLs significant in brain multi-region analysis with those in GTEx tibial nerve samples, by eGene. (XLSX)

S11 Table Effect sizes for CACNA1C *cis*-eQTLs by brain region and study. (XLSX)

S12 Table ZNF804A *cis*-eQTL effect sizes by region and study. (XLSX)

S13 Table Colocalisation analysis of schizophrenia-related GWAS SNPs. (XLSX)

S1 Data UK Brain Expression Consortium (UKBEC) members and affiliations. (PDF)
