# Supplementary figures and images for: Identification of expression quantitative trait loci associated with schizophrenia and affective disorders in normal brain tissue
Source: PLoS Genet. 2018 Aug 24;14(8):e1007607. doi: 10.1371/journal.pgen.1007607 (PMC6126875; doi:10.1371/journal.pgen.1007607)

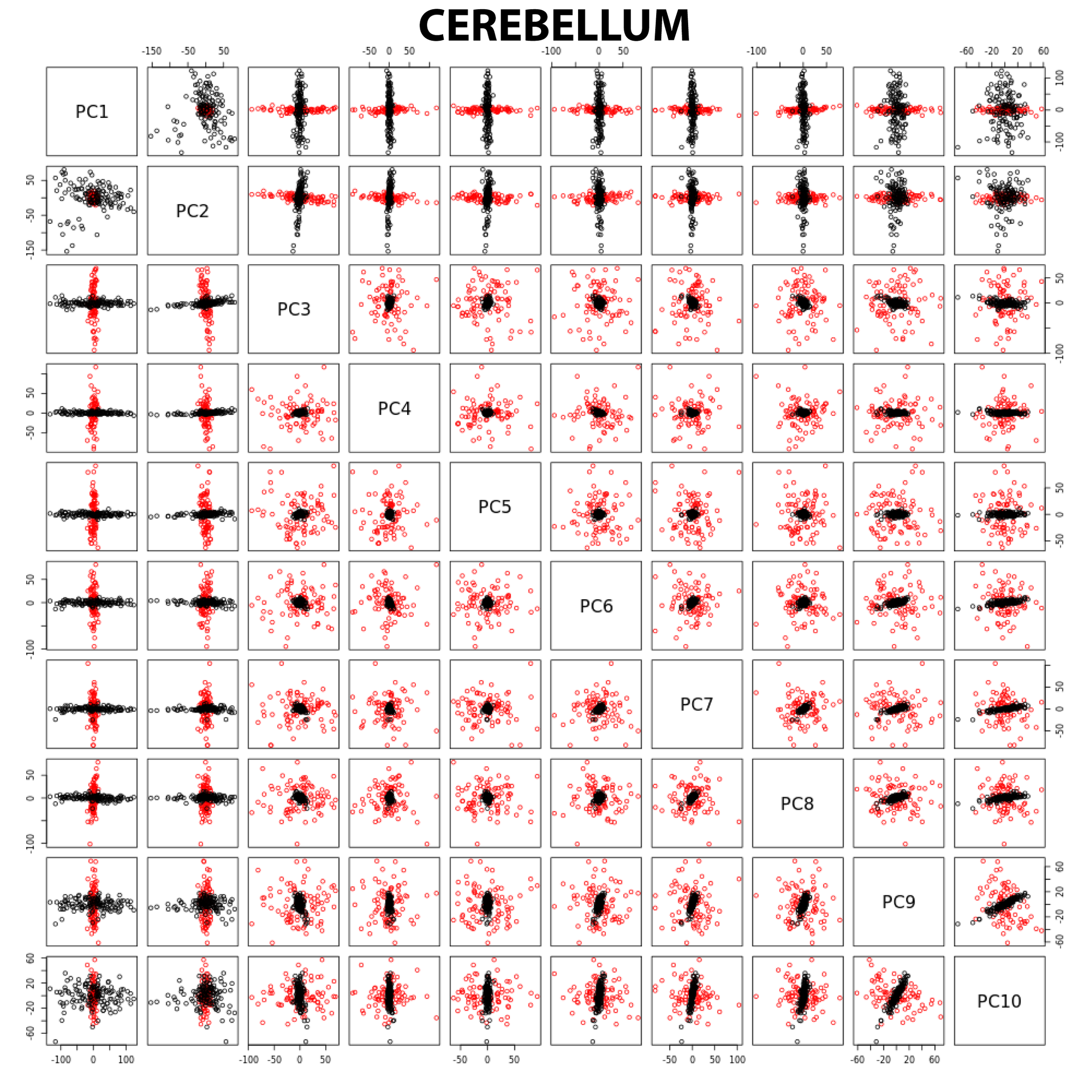

Supplement: S1 Fig — First ten principal components (PC) are shown. Red points, GTEx; black points; UKBEC. (TIF) [file pgen.1007607.s001.tif]

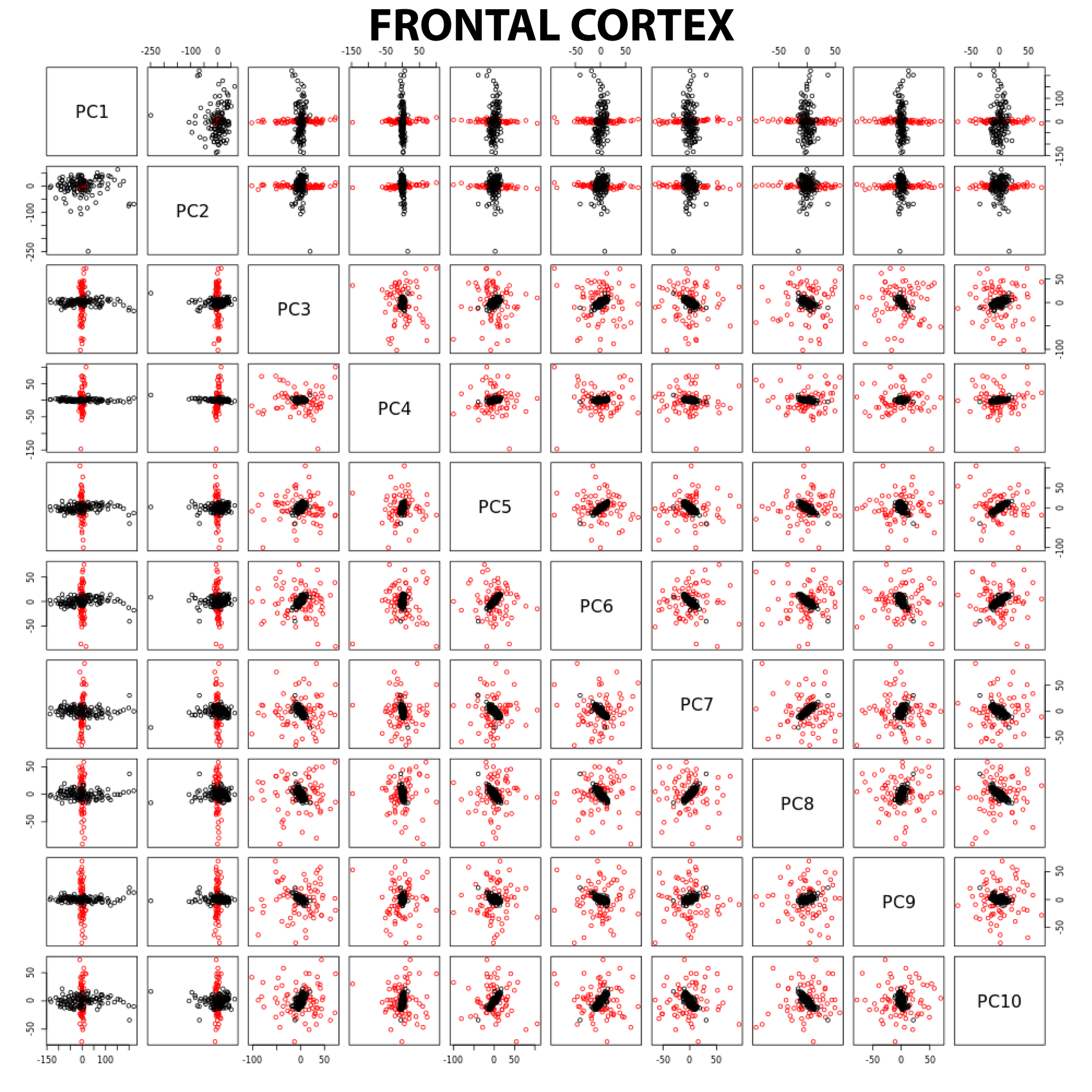

Supplement: S2 Fig — First ten principal components (PC) are shown. Red points, GTEx; black points; UKBEC. (TIF) [file pgen.1007607.s002.tif]

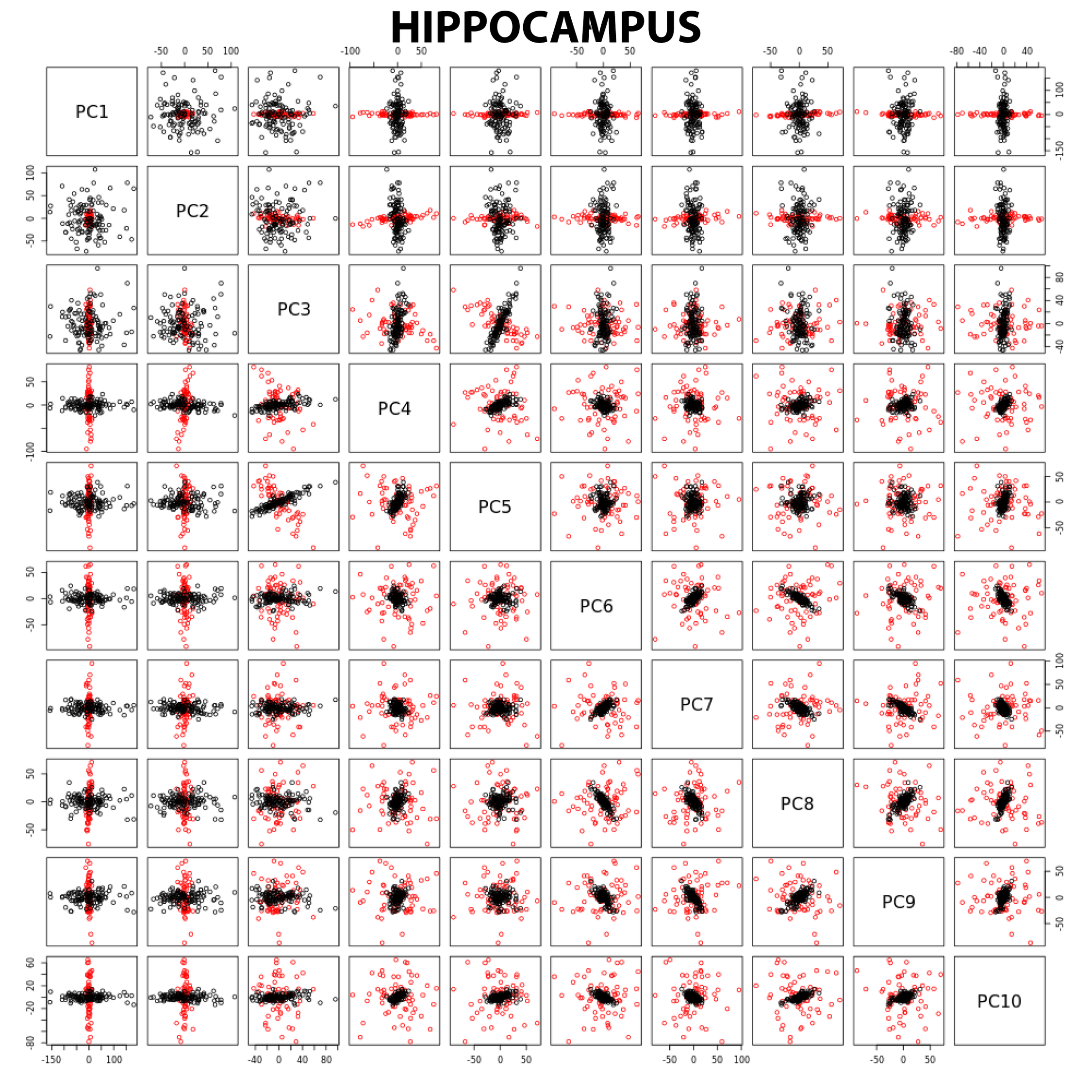

Supplement: S3 Fig — First ten principal components (PC) are shown. Red points, GTEx; black points; UKBEC. (TIF) [file pgen.1007607.s003.tif]

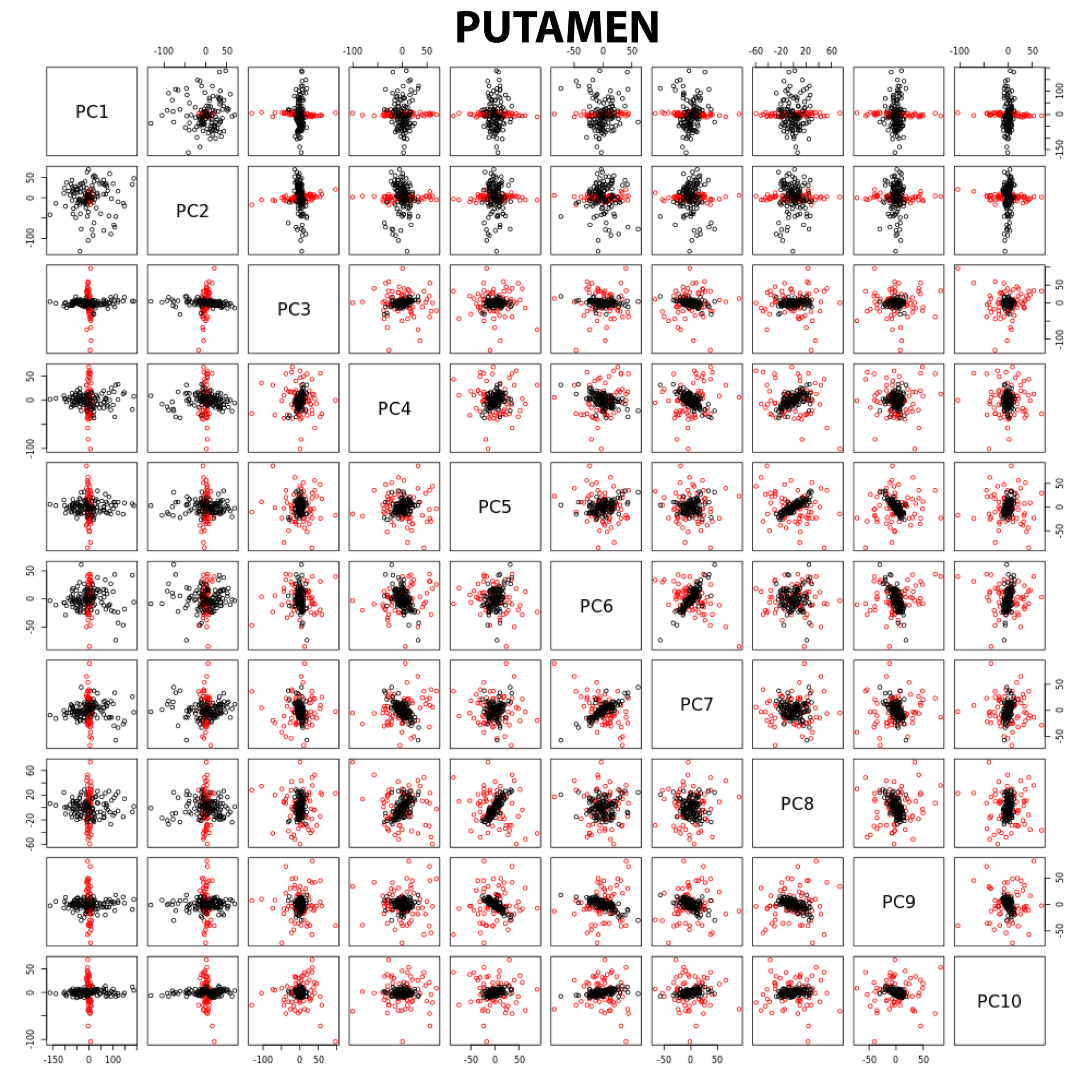

Supplement: S4 Fig — First ten principal components (PC) are shown. Red points, GTEx; black points; UKBEC. (TIF) [file pgen.1007607.s004.tif]

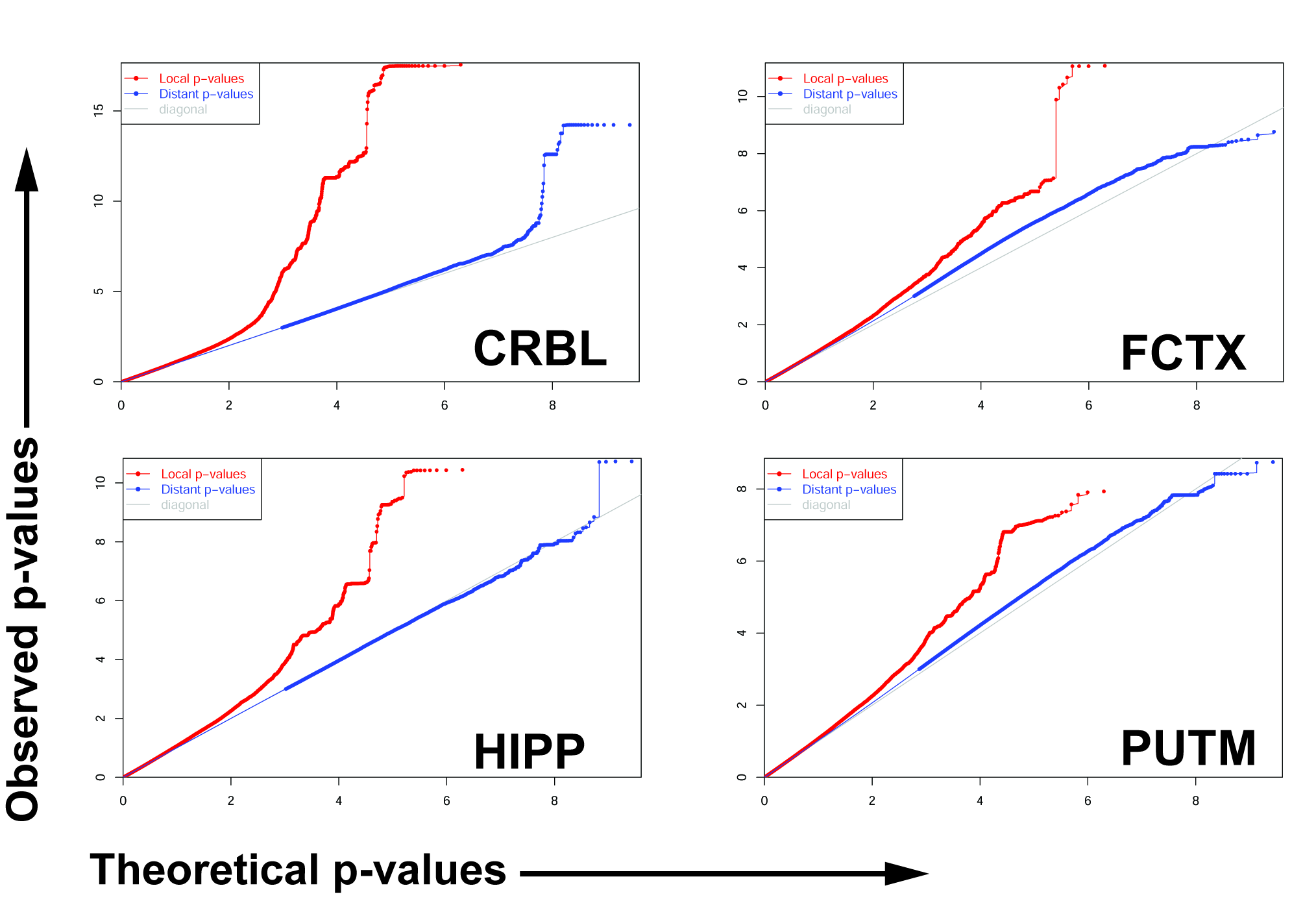

Supplement: S5 Fig — Theoretical (x-axis) versus observed (y-axis) Matrix eQTL calculated p-values (-log10) for each analysed region in UKBEC data. Red points, cis-eQTLs; blue points, trans-eQTLs; grey line represents null line. CRBL, cerebellum; FCTX, frontal cortex; HIPP, hippocampus; PUTM, putamen. (TIF) [file pgen.1007607.s005.tif]

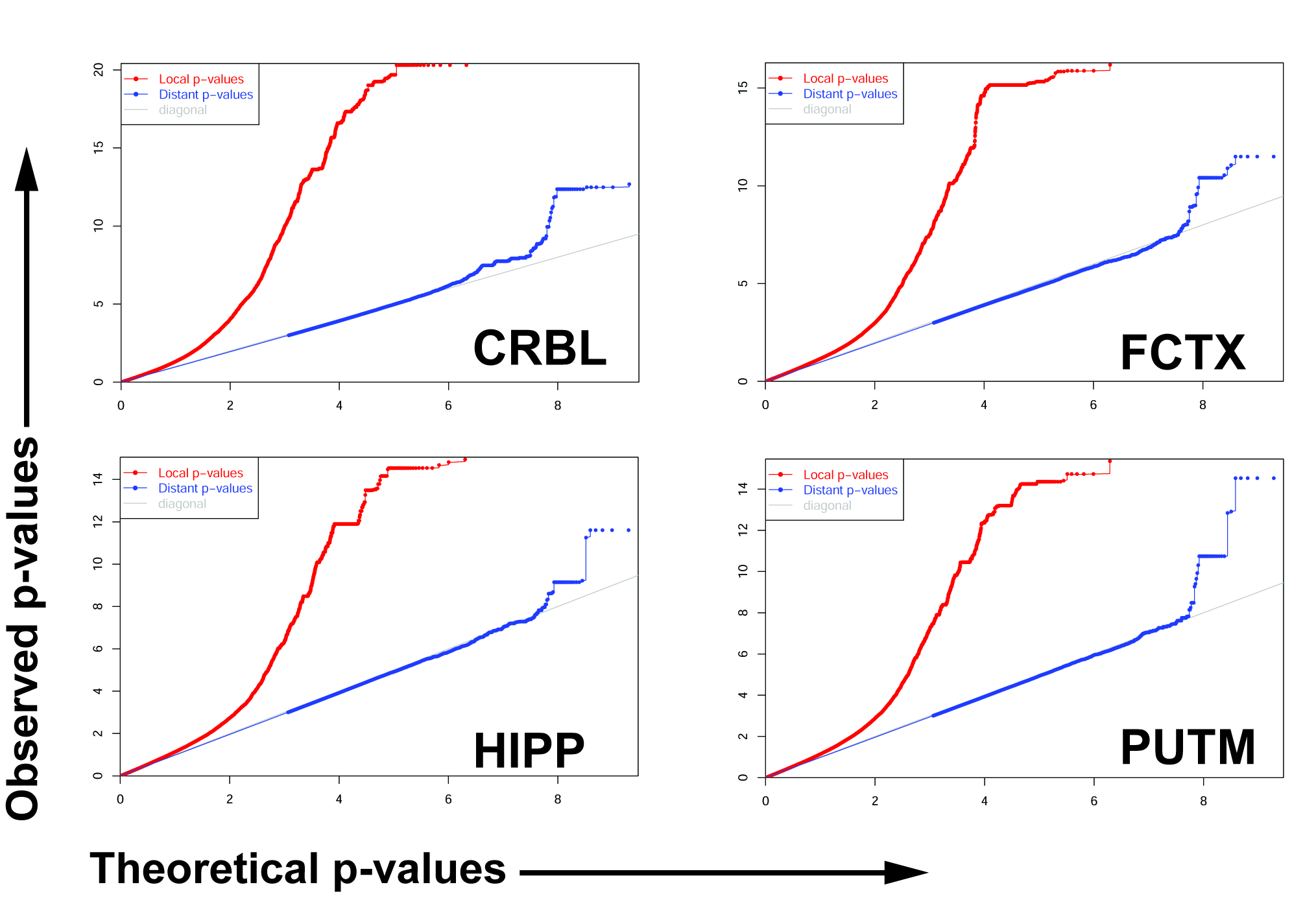

Supplement: S6 Fig — Theoretical (x-axis) versus observed (y-axis) Matrix eQTL calculated p-values (-log10) for each analysed region in GTEx data. Red points, cis-eQTLs; blue points, trans-eQTLs; grey line represents null line. See S5 Fig for abbreviations. (TIF) [file pgen.1007607.s006.tif]

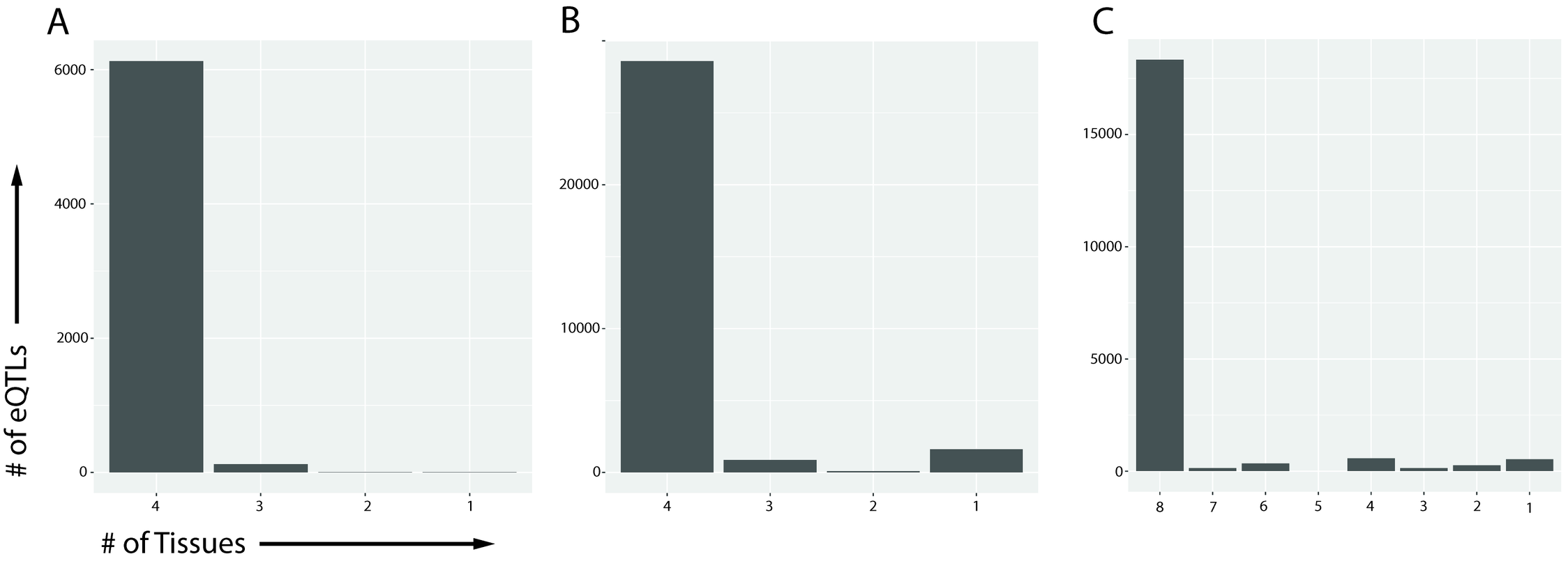

Supplement: S7 Fig — cis-eQTLs were analysed using the MT-eQTL model to identify the distribution amongst the tissues. Cerebellum, frontal cortex, hippocampus and putamen were assessed for each UKBEC (A) and GTEx (B). All eight regions were assessed for the UKBEC + GTEx analysis (C). A majority of the cis-eQTLs were present in all tissues assessed (four for UKBEC and GTEx and eight for UKBEC + GTEx). (TIF) [file pgen.1007607.s007.tif]

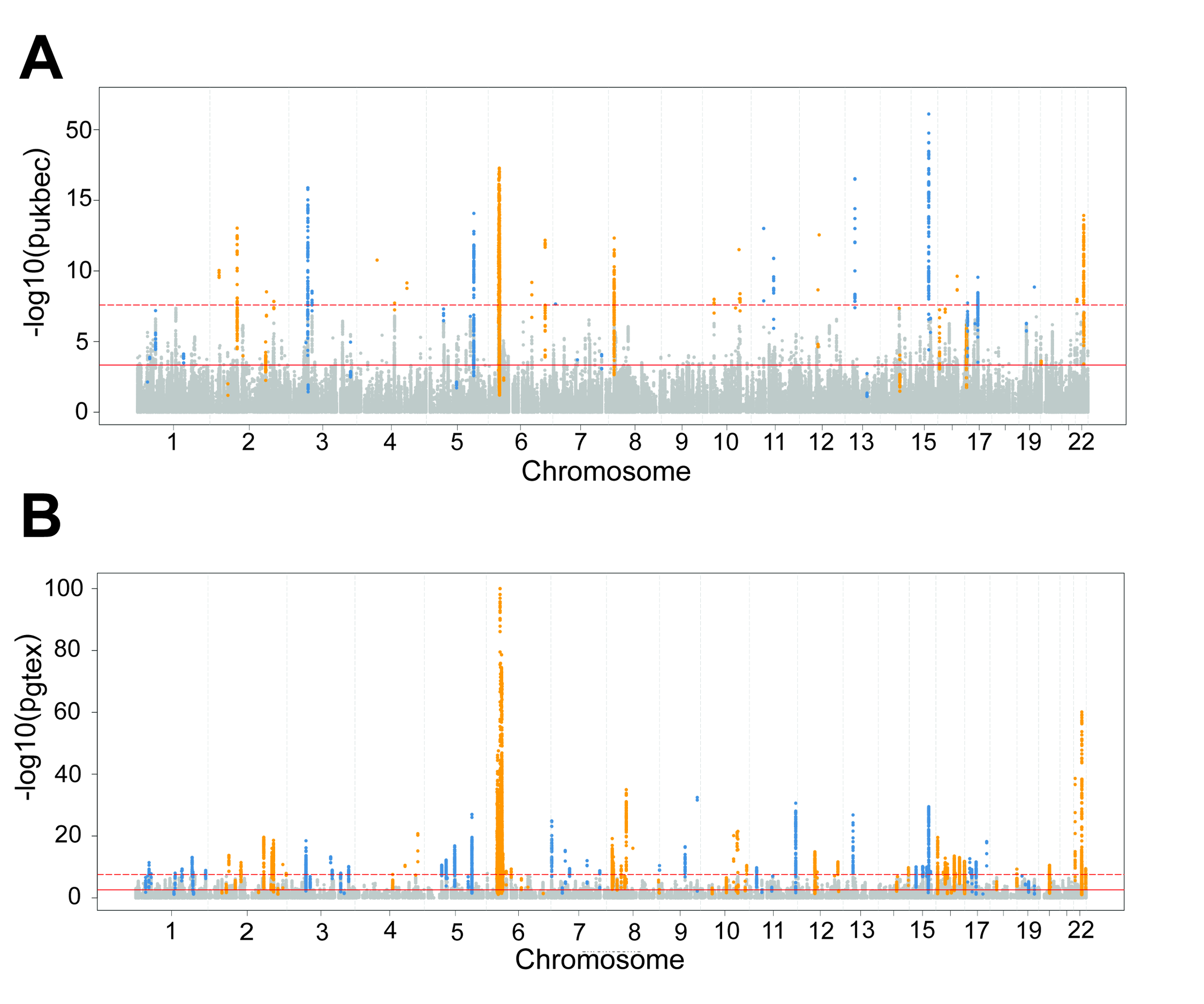

Supplement: S8 Fig — cis-eQTLs from meta-analysis of four UKBEC (A) and GTEx (B) brain regions (cerebellum, frontal cortex, hippocampus and putamen), plotted for pukbec/gtex (-log10). Colored point (blue and yellow) indicate eQTLs that are multi-region eQTLs in the four brain regions. Grey points indicate eQTLs that are not multi-region eQTLs in all four regions. Dashed red line indicates level where p-value < pbonferroni-ukbec/gtex; solid red line indicates p-value at which FDR = 0.05. Note different scales for y-axes. (TIF) [file pgen.1007607.s008.tif]

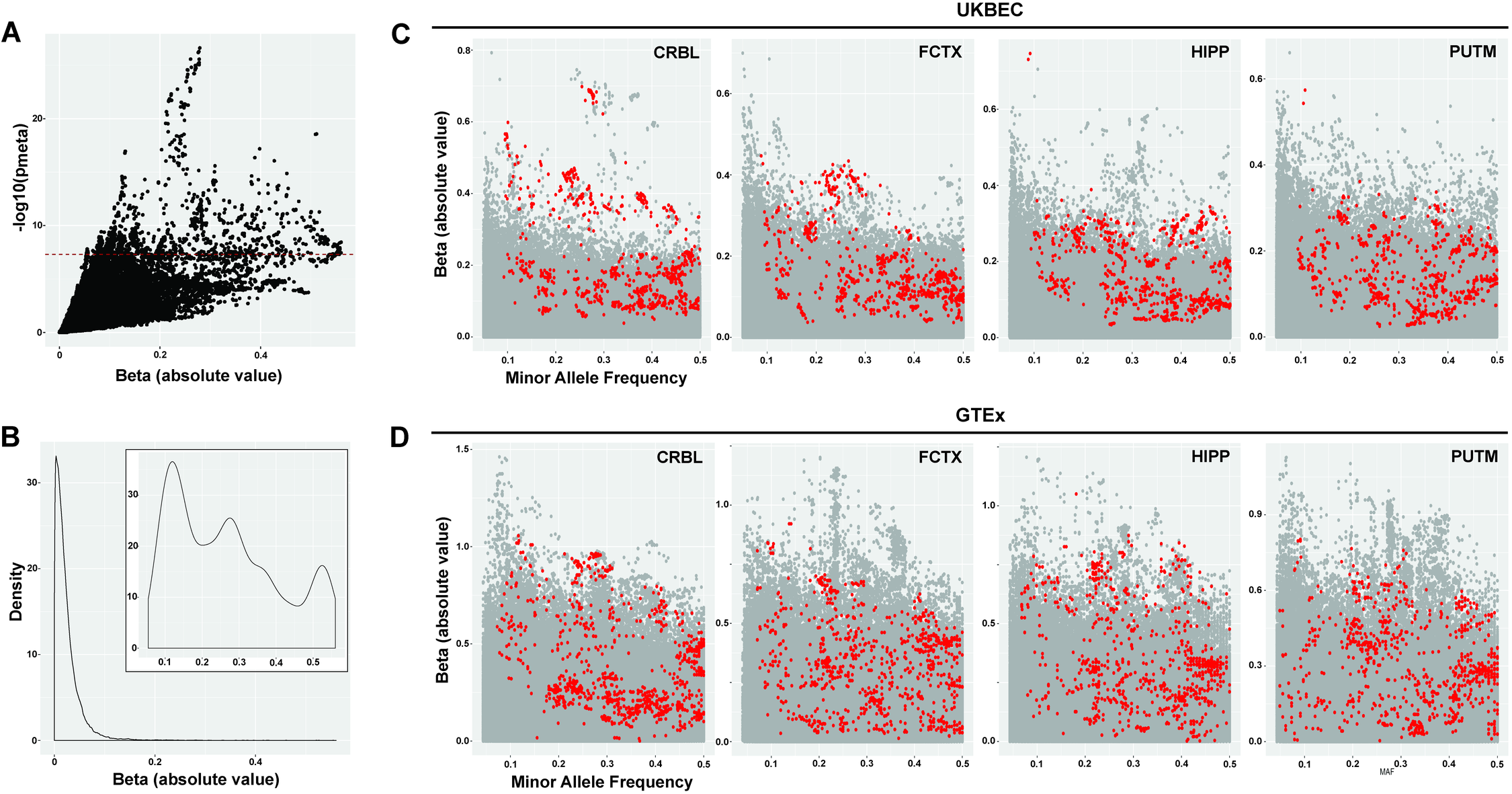

Supplement: S10 Fig — A, Absolute values of effect sizes (beta, standardised expression units per allele) plotted against pmeta (-log10) for cis-eQTLs detected in multi-region meta-analyses. Red dashed line indicates pbonferroni-meta. B, Density plot of (absolute value of) effect sizes, regardless of pmeta values. Inset depicts density plot for eQTLs with pmeta < pbonferroni-meta. C, D, cis-eQTLs plotted for minor allele frequencies (calculated per study-region) and effect sizes per region for UKBEC and GTEx, respectively. Effect sizes are based on eQTL analysis per region and study. Red colored points represent significant cis-eQTLs from multi-region meta-analyses. CRBL, cerebellum; FCTX, frontal cortex; HIPP, hippocampus; PUTM, putamen. (TIF) [file pgen.1007607.s010.tif]

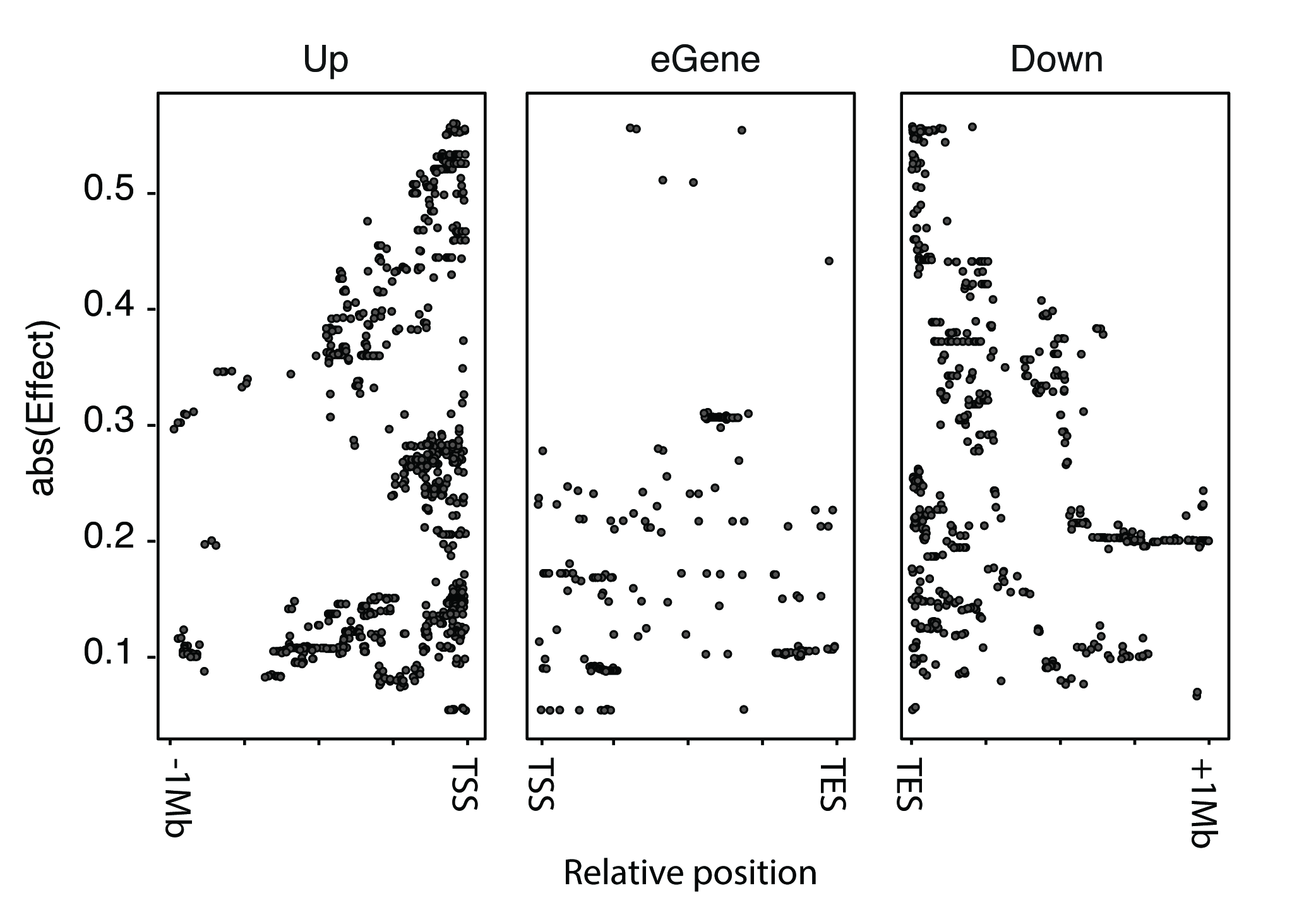

Supplement: S11 Fig — Scatter plot of absolute value of effect sizes (abs(Effect)) for the high-confidence eQTLs from the multi-region meta-analysis. Point are plotted with respect to the TSS (transcription start site) and TES (transcription end site) of the associated eGene. (TIF) [file pgen.1007607.s011.tif]

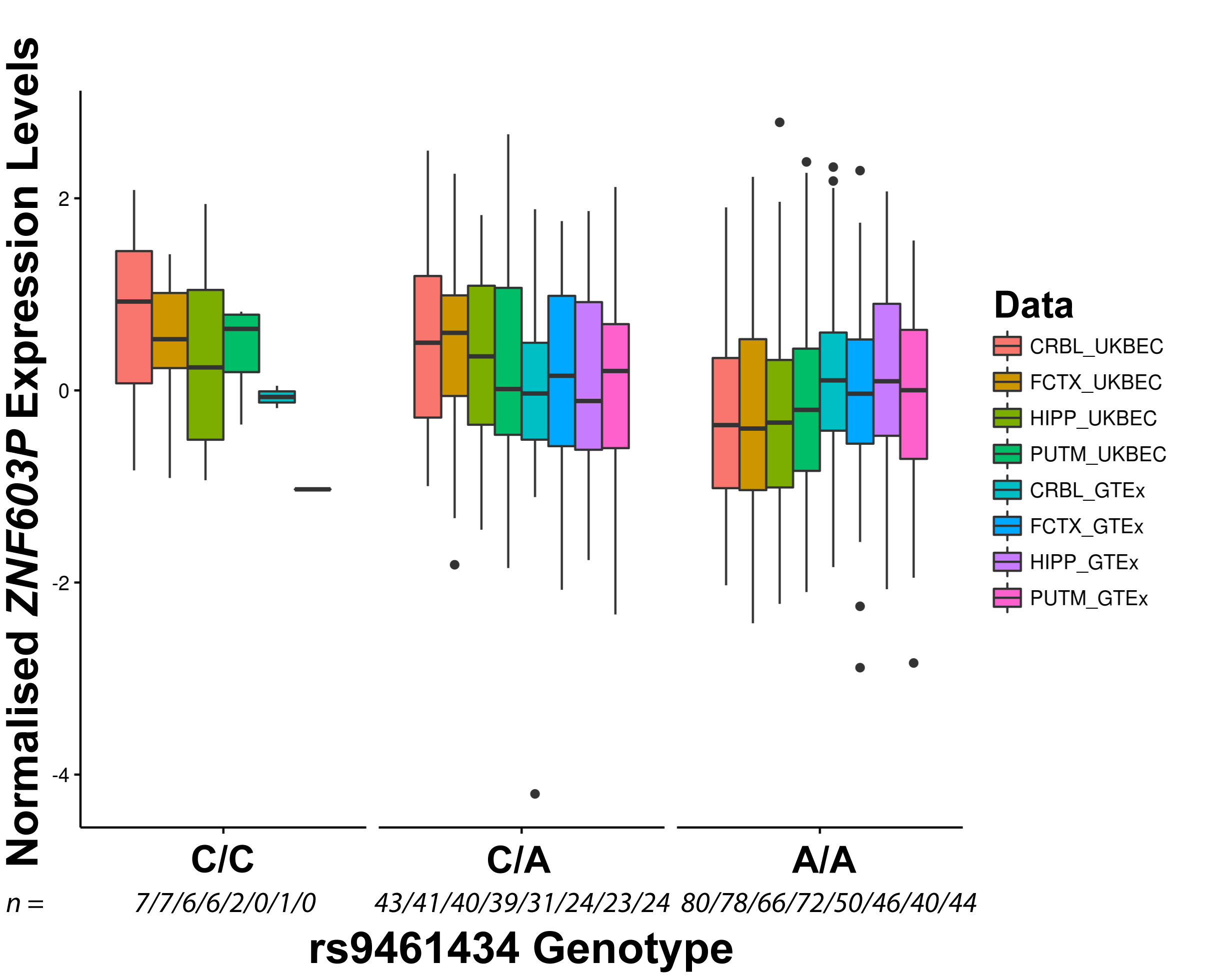

Supplement: S12 Fig — Boxplot of ZNF603P expression levels (normalised separately for UKBEC and GTEx per region) by genotype of the rs9467434 eQTL. This eQTL had the largest magnitude effect size, -0.56 ± 0.09 EU per allele for AA relative to CC, in the multi-region meta-analysis of cis-eQTLs. Vertical lines for each plot captures data between -1.5 x interquartile rage and 1.5 x interquartile range, with outliers depicted as black points. (TIF) [file pgen.1007607.s012.tif]

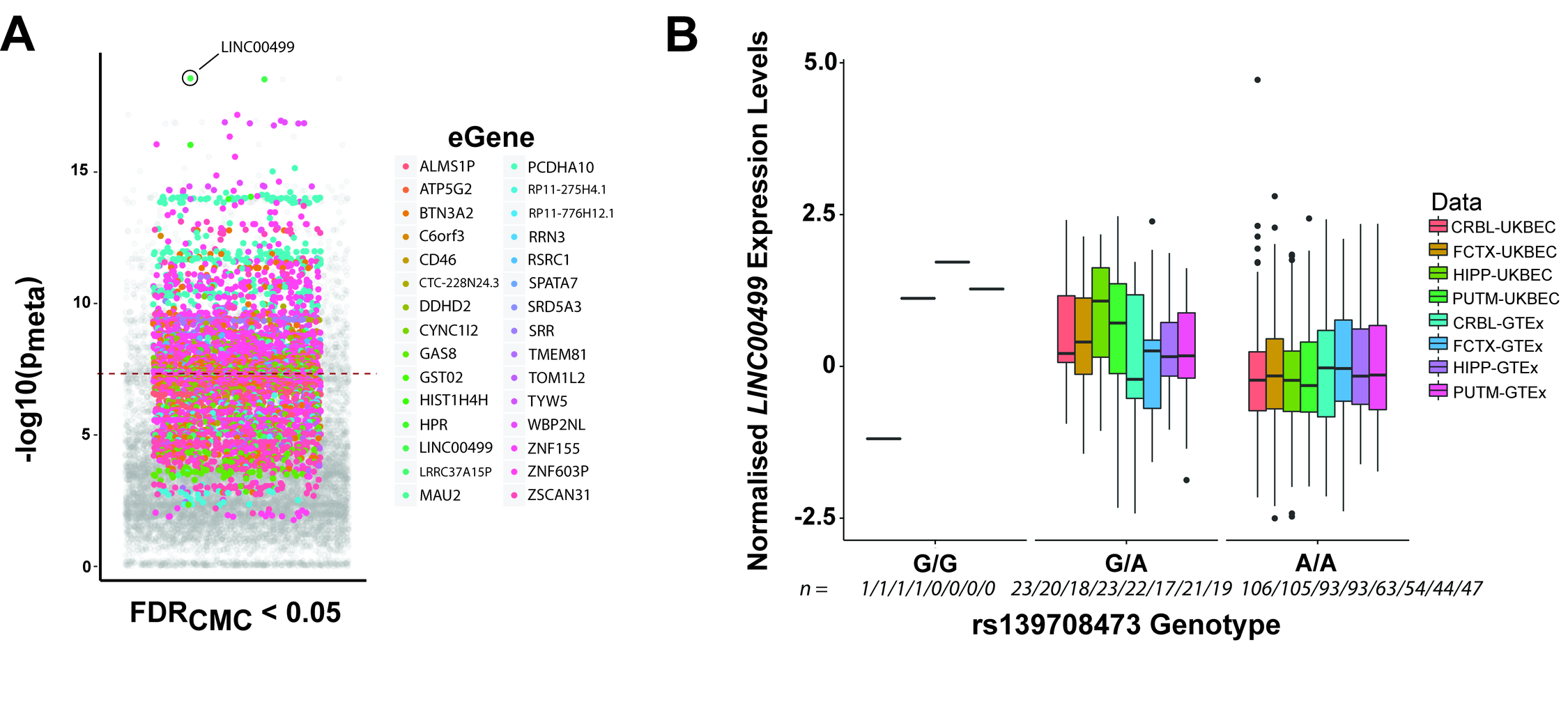

Supplement: S13 Fig — A, CMC cis-eQTLs with FDRCMC < 0.05 plotted for significance (-log10(pmeta)) from multi-region meta-analysis significant eQTLs (Fig 1C). Grey points indicate MT-eQTL meta-analysis eQTLs with FDRmeta < 0.05 and present in all regions studied. Colored eQTLs, by eGene, represent those that are associated with eGenes from the high-confidence list (see main text). Red line indicates pbonferroni threshold for the UKBEC+GTEx meta-analysis. B, Boxplot of LINC00499 expression levels (normalised separately for UKBEC and GTEx per region) by genotype of the rs139708473 eQTL. This eQTL had the largest magnitude effect size, -0.51 ± 0.06 EU per allele for AA relative to GG, of the multi-region meta-analysis of cis-eQTLs that overlapped with CMC. Vertical lines for each plot captures data between -1.5 x interquartile rage and 1.5 x interquartile range, with outliers depicted as black points. (TIF) [file pgen.1007607.s013.tif]

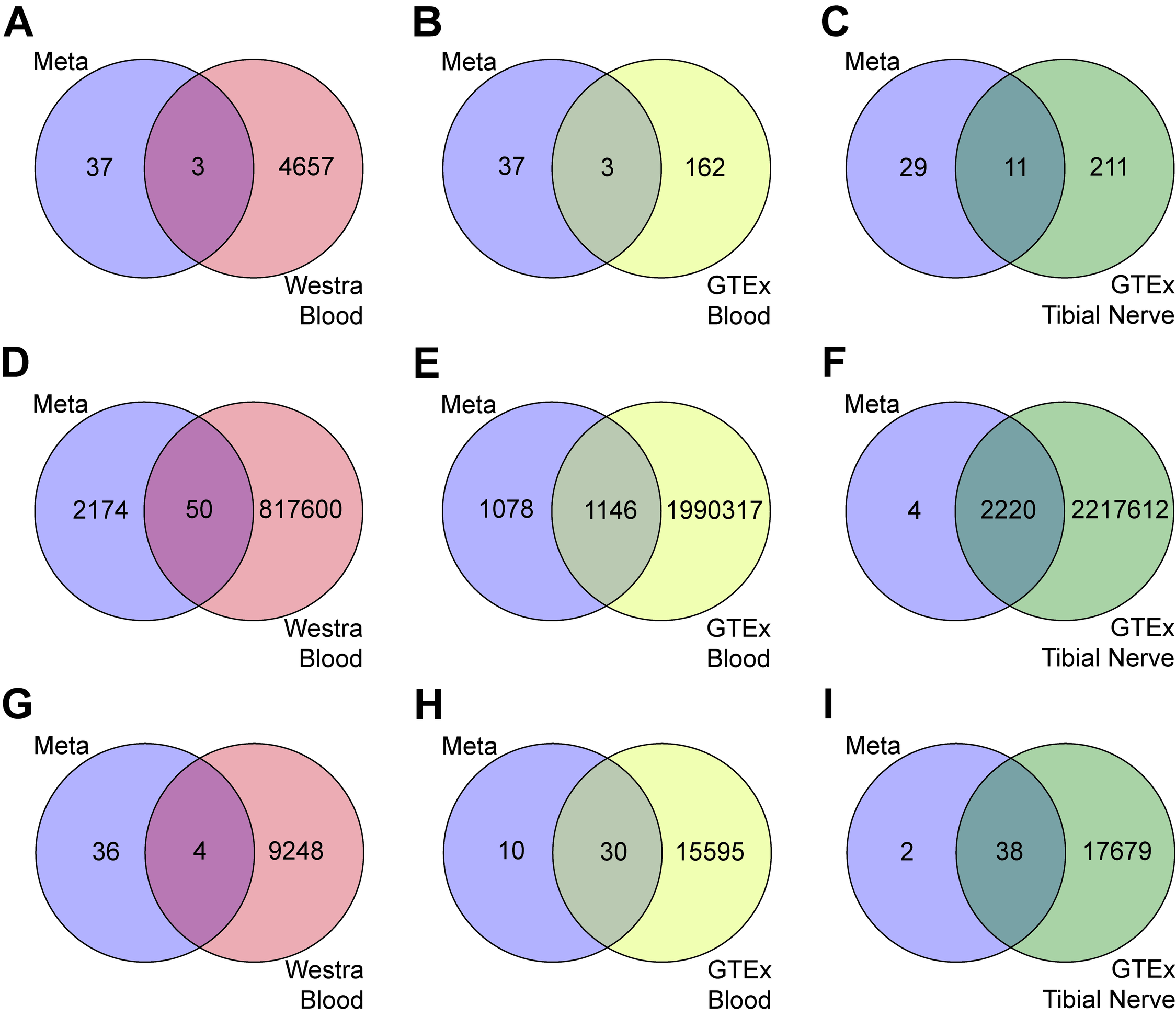

Supplement: S14 Fig — A-C, Venn diagram of eGenes associated with significant eQTLs (see Fig 4A–4C) for eQTLs from the Blood eQTL Browser (Westra Blood), GTEx whole blood and GTEx tibial nerve, respectively. D-F, Venn diagram of significant eQTLs from multi-region meta-analyses (Meta) irrespective of significance in eQTLs from Westra Blood, GTEx whole blood and GTEx tibial nerve, respectively. G-I, Number of eGenes associated with eQTLs from D-F. (TIF) [file pgen.1007607.s014.tif]

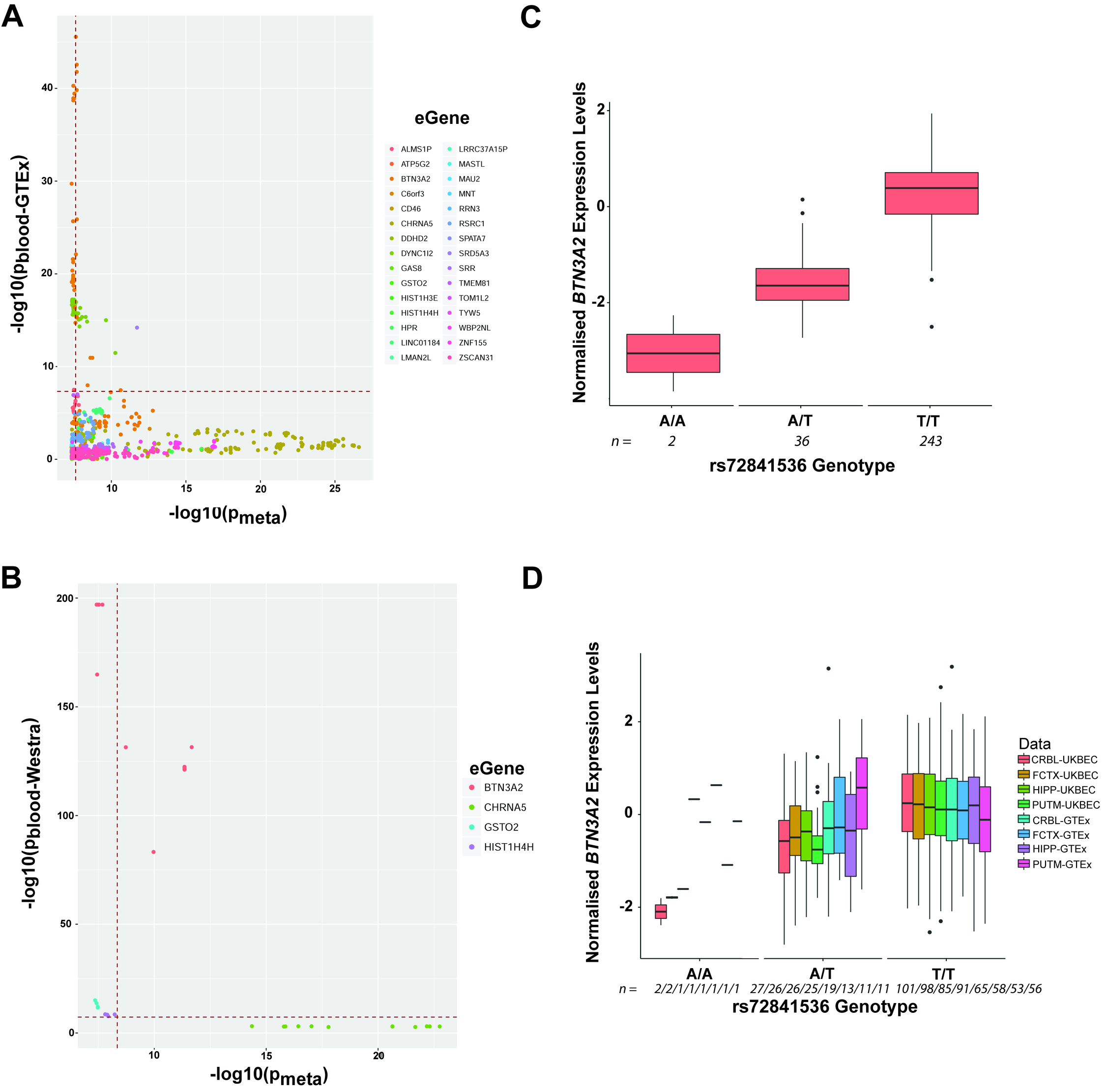

Supplement: S15 Fig — p-value plot of cis-eQTLs brain (pmeta) versus A, GTEx sample of whole blood (pblood-GTEx) or B, meta-analysis of whole blood (pblood-Westra). eQTLs are coloured by associated eGene. Dashed grey lines indicate pbonferroni-blood (horizontal line) and pbonferroni-meta (vertical line). eQTL plots for rs72841536 for BTN3A2 expression in GTEx whole blood samples (C) and in overlapping brain regions in UKBEC and GTEx samples (D). This eQTL was the most significant blood eQTL (in GTEx whole blood samples) that overlapped with cis-eQTLs identified through multi-region meta-analyses. (TIF) [file pgen.1007607.s015.tif]

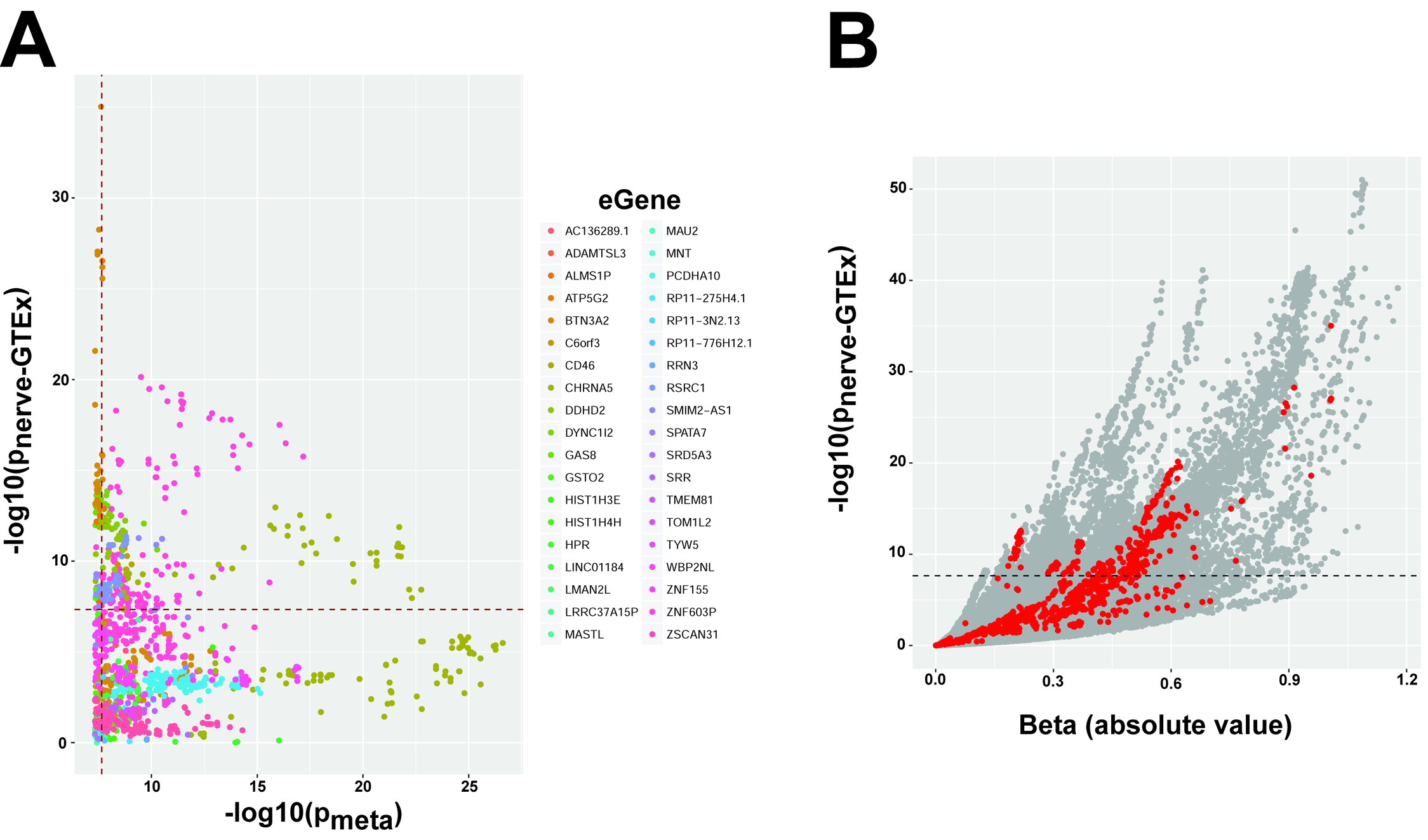

Supplement: S16 Fig — A, p-value plot of cis-eQTLs brain (pmeta) versus GTEx tibial nerve samples (pnerve-GTEx). eQTLs are coloured by associated eGene. Dashed grey lines indicate pbonferroni-meta (x-axis) and pbonferroni-tibial (y-axis). B, Absolute values of effect sizes (beta, standardised expression units per allele) plotted against pnerve-GTEx (-log10) for cis-eQTLs detected in GTEx tibial nerve samples. Red points indicate those cis-eQTLs significant in multi-region meta-analysis. Black dashed line indicates pbonferroni-tibial. (TIF) [file pgen.1007607.s016.tif]

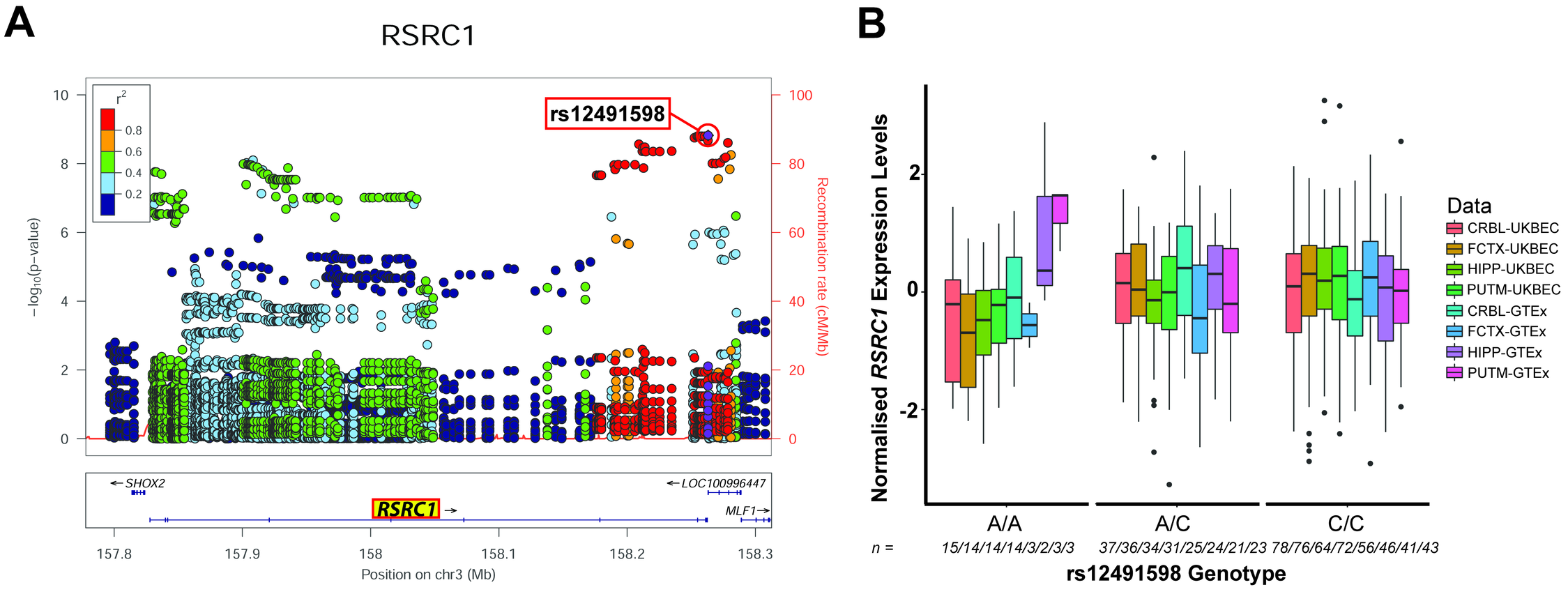

Supplement: S17 Fig — A, LocusZoom plot for the cis-eQTL rs12491598, identified in multi-region meta-analyses (Table 1). This SNP is cis to RSRC1, denoted in yellow highlight. Other SNPs that are within the study and cis to RSRC1 are plotted for their pmeta (left y-axis) and LD-value (r2 denoted by colour of circle, calculated as relative to rs12491598). Recombination rate of genomic region is plotted in red. B, Boxplot of gene expression levels (normalised separately for UKBEC and GTEx per region) by genotype of the rs12491598 eQTL. Vertical lines for each plot capture data between -1.5 x interquartile rage and 1.5 x interquartile range, with outliers depicted as black points. The number of individuals with a particular genotype per study-region is denoted below in italics. CRBL, cerebellum; FCTX, frontal cortex; HIPP, hippocampus; PUTM, putamen. (TIF) [file pgen.1007607.s017.tif]
