# Supplementary material for: Identification of expression quantitative trait loci associated with schizophrenia and affective disorders in normal brain tissue
Source: PLoS Genet. 2018 Aug 24;14(8):e1007607. doi: 10.1371/journal.pgen.1007607 (PMC6126875; doi:10.1371/journal.pgen.1007607)
Supplement: S9 Fig — A-MO, Forest plots, by study, of 41 cis-eQTLs through multi-region meta-analyses. Each plot is denoted by the eQTL and corresponding eGene (i.e. rs139708473 –LINC00499 for A). Note that summarised forest plots for cis-eQTLs rs12438181 –CHRNA5 (MN) and rs16969968 –CHRNA5 (MO) are shown in Fig 3B and 3C. C, cerebellum; F, frontal cortex; H, hippocampus; P, putamen. (PDF) [file pgen.1007607.s009.pdf]

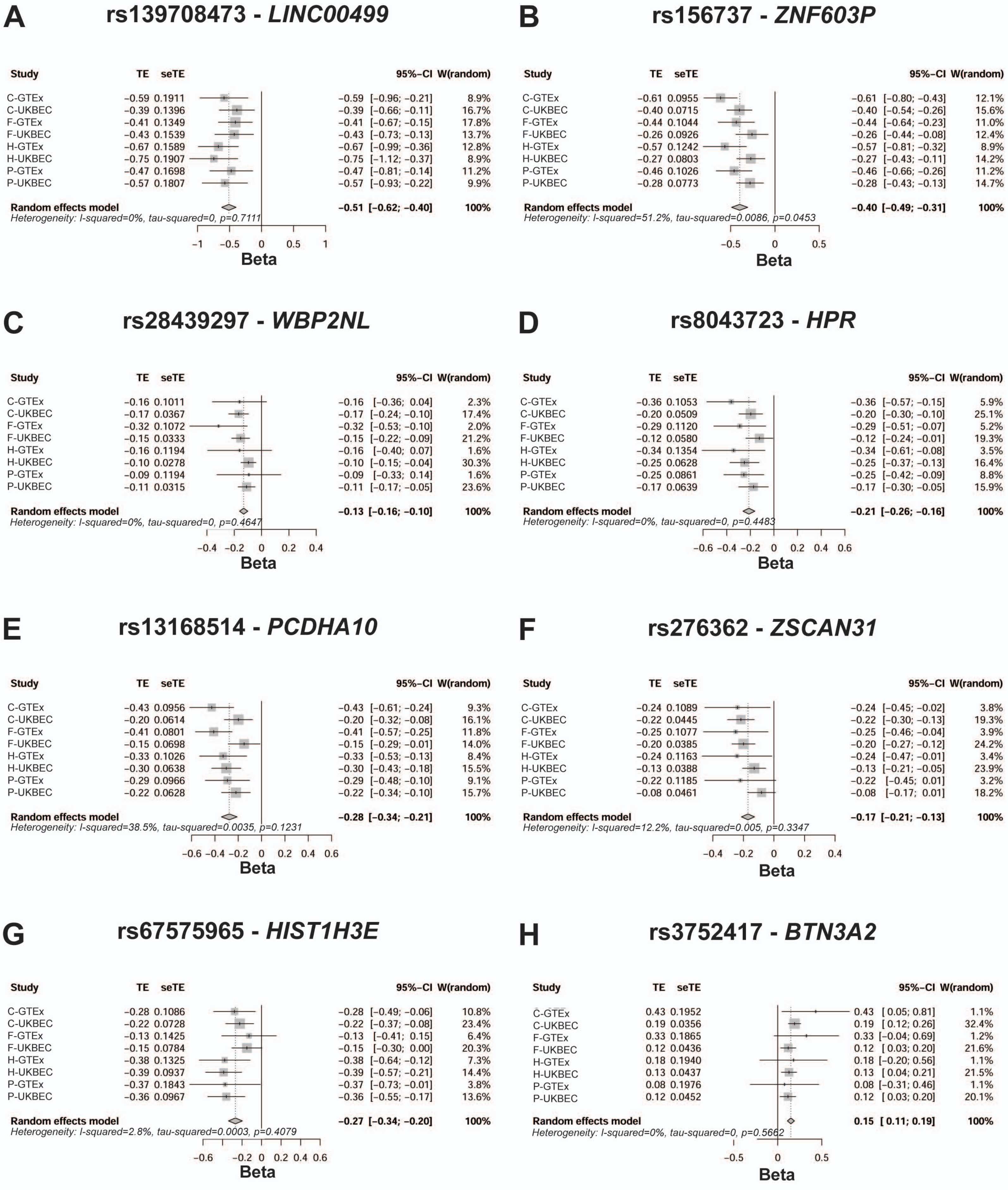
A
rs139708473 - LINC00499
Study TE seTE 95%-CI W(random)
C-GTEx −0.59 0.1911 −0.59 [−0.96; −0.21] 8.9%
C-UKBEC −0.39 0.1396 −0.39 [−0.66; −0.11] 16.7%
F-GTEx −0.41 0.1349 −0.41 [−0.67; −0.15] 17.8%
F-UKBEC −0.43 0.1539 −0.43 [−0.73; −0.13] 13.7%
H-GTEx −0.67 0.1589 −0.67 [−0.99; −0.36] 12.8%
H-UKBEC −0.75 0.1907 −0.75 [−1.12; −0.37] 8.9%
P-GTEx −0.47 0.1698 −0.47 [−0.81; −0.14] 11.2%
P-UKBEC −0.57 0.1807 −0.57 [−0.93; −0.22] 9.9%
Random effects model −0.51 [−0.62; −0.40] 100%
Heterogeneity: I-squared=0%, tau-squared=0, p=0.7111
−1 −0.5 0 0.5 1
Beta
B
rs156737 - ZNF603P
Study TE seTE 95%-CI W(random)
C-GTEx −0.61 0.0955 −0.61 [−0.80; −0.43] 12.1%
C-UKBEC −0.40 0.0715 −0.40 [−0.54; −0.26] 15.6%
F-GTEx −0.44 0.1044 −0.44 [−0.64; −0.23] 11.0%
F-UKBEC −0.26 0.0926 −0.26 [−0.44; −0.08] 12.4%
H-GTEx −0.57 0.1242 −0.57 [−0.81; −0.32] 8.9%
H-UKBEC −0.27 0.0803 −0.27 [−0.43; −0.11] 14.2%
P-GTEx −0.46 0.1026 −0.46 [−0.66; −0.26] 11.2%
P-UKBEC −0.28 0.0773 −0.28 [−0.43; −0.13] 14.7%
Random effects model −0.40 [−0.49; −0.31] 100%
Heterogeneity: I-squared=51.2%, tau-squared=0.0086, p=0.0453
−0.5 0 0.5
Beta
C
rs28439297 - WBP2NL
Study TE seTE 95%-CI W(random)
C-GTEx −0.16 0.1011 −0.16 [−0.36; 0.04] 2.3%
C-UKBEC −0.17 0.0367 −0.17 [−0.24; −0.10] 17.4%
F-GTEx −0.32 0.1072 −0.32 [−0.53; −0.10] 2.0%
F-UKBEC −0.15 0.0333 −0.15 [−0.22; −0.09] 21.2%
H-GTEx −0.16 0.1194 −0.16 [−0.40; 0.07] 1.6%
H-UKBEC −0.10 0.0278 −0.10 [−0.15; −0.04] 30.3%
P-GTEx −0.09 0.1194 −0.09 [−0.33; 0.14] 1.6%
P-UKBEC −0.11 0.0315 −0.11 [−0.17; −0.05] 23.6%
Random effects model −0.13 [−0.16; −0.10] 100%
Heterogeneity: I-squared=0%, tau-squared=0, p=0.4647
−0.4 −0.2 0 0.2 0.4
Beta
D
rs8043723 - HPR
Study TE seTE 95%-CI W(random)
C-GTEx −0.36 0.1053 −0.36 [−0.57; −0.15] 5.9%
C-UKBEC −0.20 0.0509 −0.20 [−0.30; −0.10] 25.1%
F-GTEx −0.29 0.1120 −0.29 [−0.51; −0.07] 5.2%
F-UKBEC −0.12 0.0580 −0.12 [−0.24; −0.01] 19.3%
H-GTEx −0.34 0.1354 −0.34 [−0.61; −0.08] 3.5%
H-UKBEC −0.25 0.0628 −0.25 [−0.37; −0.13] 16.4%
P-GTEx −0.25 0.0861 −0.25 [−0.42; −0.09] 8.8%
P-UKBEC −0.17 0.0639 −0.17 [−0.30; −0.05] 15.9%
Random effects model −0.21 [−0.26; −0.16] 100%
Heterogeneity: I-squared=0%, tau-squared=0, p=0.4483
−0.6 −0.4 −0.2 0 0.2 0.4 0.6
Beta
E
rs13168514 - PCDHA10
Study TE seTE 95%-CI W(random)
C-GTEx −0.43 0.0956 −0.43 [−0.61; −0.24] 9.3%
C-UKBEC −0.20 0.0614 −0.20 [−0.32; −0.08] 16.1%
F-GTEx −0.41 0.0801 −0.41 [−0.57; −0.25] 11.8%
F-UKBEC −0.15 0.0698 −0.15 [−0.29; −0.01] 14.0%
H-GTEx −0.33 0.1026 −0.33 [−0.53; −0.13] 8.4%
H-UKBEC −0.30 0.0638 −0.30 [−0.43; −0.18] 15.5%
P-GTEx −0.29 0.0966 −0.29 [−0.48; −0.10] 9.1%
P-UKBEC −0.22 0.0628 −0.22 [−0.34; −0.10] 15.7%
Random effects model −0.28 [−0.34; −0.21] 100%
Heterogeneity: I-squared=38.5%, tau-squared=0.0035, p=0.1231
−0.6 −0.4 −0.2 0 0.2 0.4 0.6
Beta
F
rs276362 - ZSCAN31
Study TE seTE 95%-CI W(random)
C-GTEx −0.24 0.1089 −0.24 [−0.45; −0.02] 3.8%
C-UKBEC −0.22 0.0445 −0.22 [−0.30; −0.13] 19.3%
F-GTEx −0.25 0.1077 −0.25 [−0.46; −0.04] 3.9%
F-UKBEC −0.20 0.0385 −0.20 [−0.27; −0.12] 24.2%
H-GTEx −0.24 0.1163 −0.24 [−0.47; −0.01] 3.4%
H-UKBEC −0.13 0.0388 −0.13 [−0.21; −0.05] 23.9%
P-GTEx −0.22 0.1185 −0.22 [−0.45; 0.01] 3.2%
P-UKBEC −0.08 0.0461 −0.08 [−0.17; 0.01] 18.2%
Random effects model −0.17 [−0.21; −0.13] 100%
Heterogeneity: I-squared=12.2%, tau-squared=0.005, p=0.3347
−0.4 −0.2 0 0.2 0.4
Beta
G
rs67575965 - HIST1H3E
Study TE seTE 95%-CI W(random)
C-GTEx −0.28 0.1086 −0.28 [−0.49; −0.06] 10.8%
C-UKBEC −0.22 0.0728 −0.22 [−0.37; −0.08] 23.4%
F-GTEx −0.13 0.1425 −0.13 [−0.41; 0.15] 6.4%
F-UKBEC −0.15 0.0784 −0.15 [−0.30; 0.00] 20.3%
H-GTEx −0.38 0.1325 −0.38 [−0.64; −0.12] 7.3%
H-UKBEC −0.39 0.0937 −0.39 [−0.57; −0.21] 14.4%
P-GTEx −0.37 0.1843 −0.37 [−0.73; −0.01] 3.8%
P-UKBEC −0.36 0.0967 −0.36 [−0.55; −0.17] 13.6%
Random effects model −0.27 [−0.34; −0.20] 100%
Heterogeneity: I-squared=2.8%, tau-squared=0.0003, p=0.4079
−0.6 −0.2 0 0.2 0.4 0.6
Beta
H
rs3752417 - BTN3A2
Study TE seTE 95%-CI W(random)
C-GTEx 0.43 0.1952 0.43 [0.05; 0.81] 1.1%
C-UKBEC 0.19 0.0356 0.19 [0.12; 0.26] 32.4%
F-GTEx 0.33 0.1865 0.33 [−0.04; 0.69] 1.2%
F-UKBEC 0.12 0.0436 0.12 [0.03; 0.20] 21.6%
H-GTEx 0.18 0.1940 0.18 [−0.20; 0.56] 1.1%
H-UKBEC 0.13 0.0437 0.13 [0.04; 0.21] 21.5%
P-GTEx 0.08 0.1976 0.08 [−0.31; 0.46] 1.1%
P-UKBEC 0.12 0.0452 0.12 [0.03; 0.20] 20.1%
Random effects model 0.15 [0.11; 0.19] 100%
Heterogeneity: I-squared=0%, tau-squared=0, p=0.5662
−0.5 0 0.5
Beta

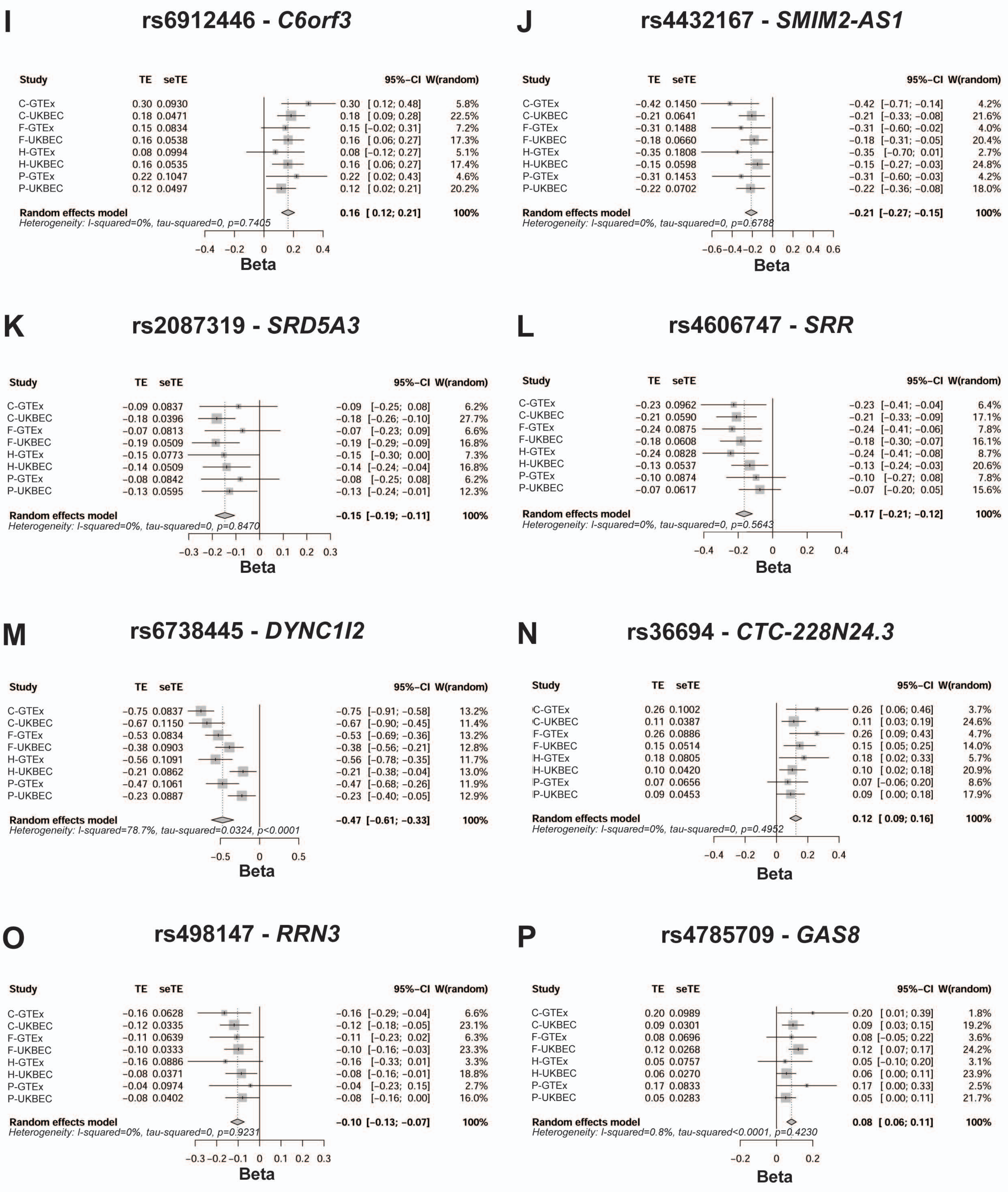
I
rs6912446 - C6orf3
Study TE seTE 95%−CI W(random)
C-GTEx 0.30 0.0930 0.30 [0.12; 0.48] 5.8%
C-UKBEC 0.18 0.0471 0.18 [0.09; 0.28] 22.5%
F-GTEx 0.15 0.0834 0.15 [−0.02; 0.31] 7.2%
F-UKBEC 0.16 0.0538 0.16 [0.06; 0.27] 17.3%
H-GTEx 0.08 0.0994 0.08 [−0.12; 0.27] 5.1%
H-UKBEC 0.16 0.0535 0.16 [0.06; 0.27] 17.4%
P-GTEx 0.22 0.1047 0.22 [0.02; 0.43] 4.6%
P-UKBEC 0.12 0.0497 0.12 [0.02; 0.21] 20.2%
Random effects model 0.16 [0.12; 0.21] 100%
Heterogeneity: I-squared=0%, tau-squared=0, p=0.7405
−0.4 −0.2 0 0.2 0.4
Beta
J
rs4432167 - SMIM2-AS1
Study TE seTE 95%−CI W(random)
C-GTEx −0.42 0.1450 −0.42 [−0.71; −0.14] 4.2%
C-UKBEC −0.21 0.0641 −0.21 [−0.33; −0.08] 21.6%
F-GTEx −0.31 0.1488 −0.31 [−0.60; −0.02] 4.0%
F-UKBEC −0.18 0.0660 −0.18 [−0.31; −0.05] 20.4%
H-GTEx −0.35 0.1808 −0.35 [−0.70; 0.01] 2.7%
H-UKBEC −0.15 0.0598 −0.15 [−0.27; −0.03] 24.8%
P-GTEx −0.31 0.1453 −0.31 [−0.60; −0.03] 4.2%
P-UKBEC −0.22 0.0702 −0.22 [−0.36; −0.08] 18.0%
Random effects model −0.21 [−0.27; −0.15] 100%
Heterogeneity: I-squared=0%, tau-squared=0, p=0.6788
−0.6 −0.4 −0.2 0 0.2 0.4 0.6
Beta
K
rs2087319 - SRD5A3
Study TE seTE 95%−CI W(random)
C-GTEx −0.09 0.0837 −0.09 [−0.25; 0.08] 6.2%
C-UKBEC −0.18 0.0396 −0.18 [−0.26; −0.10] 27.7%
F-GTEx −0.07 0.0813 −0.07 [−0.23; 0.09] 6.6%
F-UKBEC −0.19 0.0509 −0.19 [−0.29; −0.09] 16.8%
H-GTEx −0.15 0.0773 −0.15 [−0.30; 0.00] 7.3%
H-UKBEC −0.14 0.0509 −0.14 [−0.24; −0.04] 16.8%
P-GTEx −0.08 0.0842 −0.08 [−0.25; 0.08] 6.2%
P-UKBEC −0.13 0.0595 −0.13 [−0.24; −0.01] 12.3%
Random effects model −0.15 [−0.19; −0.11] 100%
Heterogeneity: I-squared=0%, tau-squared=0, p=0.8470
−0.3 −0.2 −0.1 0 0.1 0.2 0.3
Beta
L
rs4606747 - SRR
Study TE seTE 95%−CI W(random)
C-GTEx −0.23 0.0962 −0.23 [−0.41; −0.04] 6.4%
C-UKBEC −0.21 0.0590 −0.21 [−0.33; −0.09] 17.1%
F-GTEx −0.24 0.0875 −0.24 [−0.41; −0.06] 7.8%
F-UKBEC −0.18 0.0608 −0.18 [−0.30; −0.07] 16.1%
H-GTEx −0.24 0.0828 −0.24 [−0.41; −0.08] 8.7%
H-UKBEC −0.13 0.0537 −0.13 [−0.24; −0.03] 20.6%
P-GTEx −0.10 0.0874 −0.10 [−0.27; 0.08] 7.8%
P-UKBEC −0.07 0.0617 −0.07 [−0.20; 0.05] 15.6%
Random effects model −0.17 [−0.21; −0.12] 100%
Heterogeneity: I-squared=0%, tau-squared=0, p=0.5643
−0.4 −0.2 0 0.2 0.4
Beta
M
rs6738445 - DYNC1I2
Study TE seTE 95%−CI W(random)
C-GTEx −0.75 0.0837 −0.75 [−0.91; −0.58] 13.2%
C-UKBEC −0.67 0.1150 −0.67 [−0.90; −0.45] 11.4%
F-GTEx −0.53 0.0834 −0.53 [−0.69; −0.36] 13.2%
F-UKBEC −0.38 0.0903 −0.38 [−0.56; −0.21] 12.8%
H-GTEx −0.56 0.1091 −0.56 [−0.78; −0.35] 11.7%
H-UKBEC −0.21 0.0862 −0.21 [−0.38; −0.04] 13.0%
P-GTEx −0.47 0.1061 −0.47 [−0.68; −0.26] 11.9%
P-UKBEC −0.23 0.0887 −0.23 [−0.40; −0.05] 12.9%
Random effects model −0.47 [−0.61; −0.33] 100%
Heterogeneity: I-squared=78.7%, tau-squared=0.0324, p<0.0001
−0.5 0 0.5
Beta
N
rs36694 - CTC-228N24.3
Study TE seTE 95%−CI W(random)
C-GTEx 0.26 0.1002 0.26 [0.06; 0.46] 3.7%
C-UKBEC 0.11 0.0387 0.11 [0.03; 0.19] 24.6%
F-GTEx 0.26 0.0886 0.26 [0.09; 0.43] 4.7%
F-UKBEC 0.15 0.0514 0.15 [0.05; 0.25] 14.0%
H-GTEx 0.18 0.0805 0.18 [0.02; 0.33] 5.7%
H-UKBEC 0.10 0.0420 0.10 [0.02; 0.18] 20.9%
P-GTEx 0.07 0.0656 0.07 [−0.06; 0.20] 8.6%
P-UKBEC 0.09 0.0453 0.09 [0.00; 0.18] 17.9%
Random effects model 0.12 [0.09; 0.16] 100%
Heterogeneity: I-squared=0%, tau-squared=0, p=0.4952
−0.4 −0.2 0 0.2 0.4
Beta
O
rs498147 - RRN3
Study TE seTE 95%−CI W(random)
C-GTEx −0.16 0.0628 −0.16 [−0.29; −0.04] 6.6%
C-UKBEC −0.12 0.0335 −0.12 [−0.18; −0.05] 23.1%
F-GTEx −0.11 0.0639 −0.11 [−0.23; 0.02] 6.3%
F-UKBEC −0.10 0.0333 −0.10 [−0.16; −0.03] 23.3%
H-GTEx −0.16 0.0886 −0.16 [−0.33; 0.01] 3.3%
H-UKBEC −0.08 0.0371 −0.08 [−0.16; −0.01] 18.8%
P-GTEx −0.04 0.0974 −0.04 [−0.23; 0.15] 2.7%
P-UKBEC −0.08 0.0402 −0.08 [−0.16; 0.00] 16.0%
Random effects model −0.10 [−0.13; −0.07] 100%
Heterogeneity: I-squared=0%, tau-squared=0, p=0.9231
−0.3 −0.2 −0.1 0 0.1 0.2 0.3
Beta
P
rs4785709 - GAS8
Study TE seTE 95%−CI W(random)
C-GTEx 0.20 0.0989 0.20 [0.01; 0.39] 1.8%
C-UKBEC 0.09 0.0301 0.09 [0.03; 0.15] 19.2%
F-GTEx 0.08 0.0696 0.08 [−0.05; 0.22] 3.6%
F-UKBEC 0.12 0.0268 0.12 [0.07; 0.17] 24.2%
H-GTEx 0.05 0.0757 0.05 [−0.10; 0.20] 3.1%
H-UKBEC 0.06 0.0270 0.06 [0.00; 0.11] 23.9%
P-GTEx 0.17 0.0833 0.17 [0.00; 0.33] 2.5%
P-UKBEC 0.05 0.0283 0.05 [0.00; 0.11] 21.7%
Random effects model 0.08 [0.06; 0.11] 100%
Heterogeneity: I-squared=0.8%, tau-squared<0.0001, p=0.4230
−0.2 0 0.2
Beta

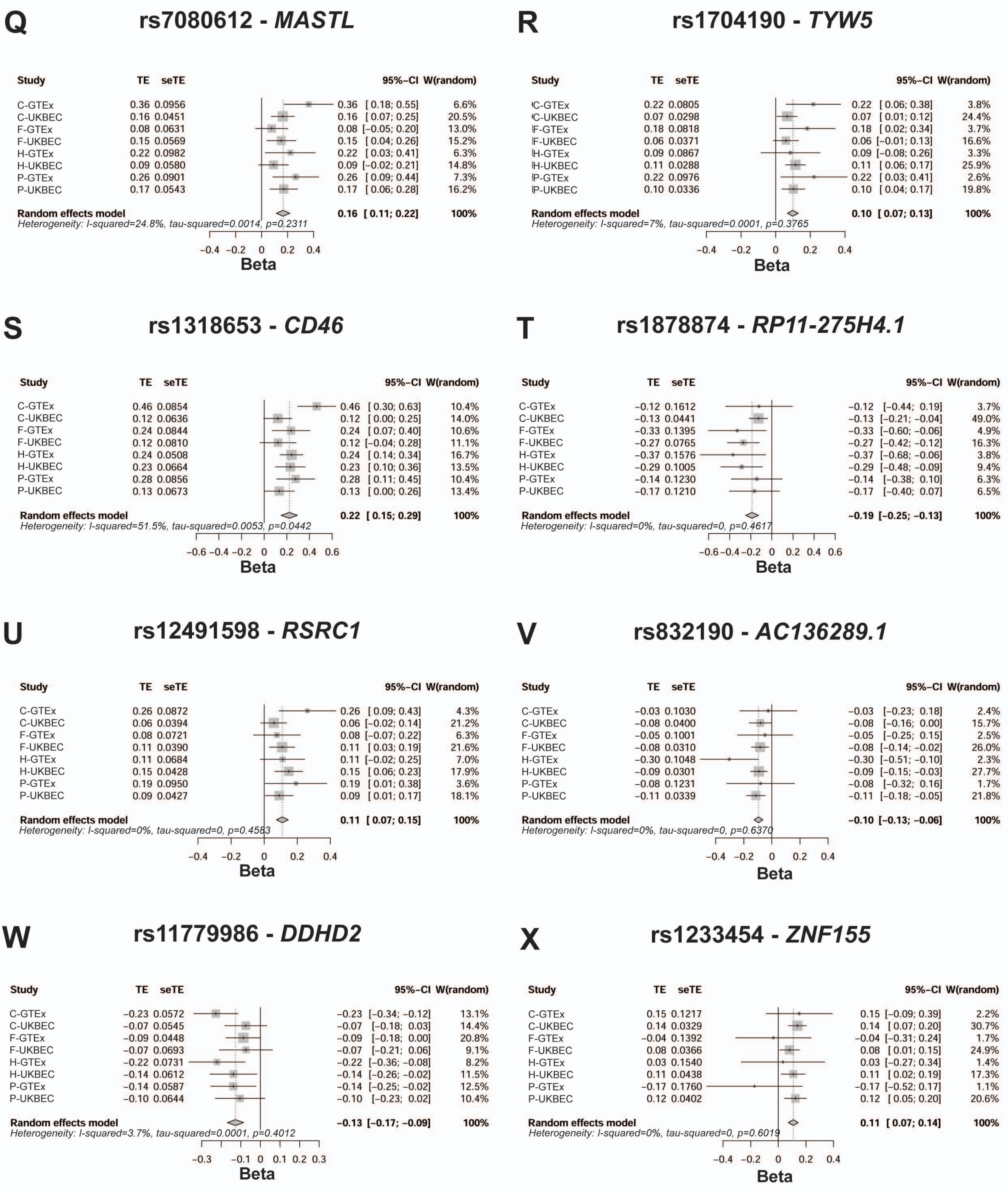
Q
rs7080612 - MASTL
Study TE seTE 95%-CI W(random)
C-GTEx 0.36 0.0956 0.36 [0.18; 0.55] 6.6%
C-UKBEC 0.16 0.0451 0.16 [0.07; 0.25] 20.5%
F-GTEx 0.08 0.0631 0.08 [−0.05; 0.20] 13.0%
F-UKBEC 0.15 0.0569 0.15 [0.04; 0.26] 15.2%
H-GTEx 0.22 0.0982 0.22 [0.03; 0.41] 6.3%
H-UKBEC 0.09 0.0580 0.09 [−0.02; 0.21] 14.8%
P-GTEx 0.26 0.0901 0.26 [0.09; 0.44] 7.3%
P-UKBEC 0.17 0.0543 0.17 [0.06; 0.28] 16.2%
Random effects model 0.16 [0.11; 0.22] 100%
Heterogeneity: I-squared=24.8%, tau-squared=0.0014, p=0.2311
−0.4 −0.2 0 0.2 0.4
Beta
R
rs1704190 - TYW5
Study TE seTE 95%-CI W(random)
C-GTEx 0.22 0.0805 0.22 [0.06; 0.38] 3.8%
C-UKBEC 0.07 0.0298 0.07 [0.01; 0.12] 24.4%
F-GTEx 0.18 0.0818 0.18 [0.02; 0.34] 3.7%
F-UKBEC 0.06 0.0371 0.06 [−0.01; 0.13] 16.6%
H-GTEx 0.09 0.0867 0.09 [−0.08; 0.26] 3.3%
H-UKBEC 0.11 0.0288 0.11 [0.06; 0.17] 25.9%
P-GTEx 0.22 0.0976 0.22 [0.03; 0.41] 2.6%
P-UKBEC 0.10 0.0336 0.10 [0.04; 0.17] 19.8%
Random effects model 0.10 [0.07; 0.13] 100%
Heterogeneity: I-squared=7%, tau-squared=0.0001, p=0.3765
−0.4 −0.2 0 0.2 0.4
Beta
S
rs1318653 - CD46
Study TE seTE 95%-CI W(random)
C-GTEx 0.46 0.0854 0.46 [0.30; 0.63] 10.4%
C-UKBEC 0.12 0.0636 0.12 [0.00; 0.25] 14.0%
F-GTEx 0.24 0.0844 0.24 [0.07; 0.40] 10.6%
F-UKBEC 0.12 0.0810 0.12 [−0.04; 0.28] 11.1%
H-GTEx 0.24 0.0508 0.24 [0.14; 0.34] 16.7%
H-UKBEC 0.23 0.0664 0.23 [0.10; 0.36] 13.5%
P-GTEx 0.28 0.0856 0.28 [0.11; 0.45] 10.4%
P-UKBEC 0.13 0.0673 0.13 [0.00; 0.26] 13.4%
Random effects model 0.22 [0.15; 0.29] 100%
Heterogeneity: I-squared=51.5%, tau-squared=0.0053, p=0.0442
−0.6 −0.4 −0.2 0 0.2 0.4 0.6
Beta
T
rs1878874 - RP11-275H4.1
Study TE seTE 95%-CI W(random)
C-GTEx −0.12 0.1612 −0.12 [−0.44; 0.19] 3.7%
C-UKBEC −0.13 0.0441 −0.13 [−0.21; −0.04] 49.0%
F-GTEx −0.33 0.1395 −0.33 [−0.60; −0.06] 4.9%
F-UKBEC −0.27 0.0765 −0.27 [−0.42; −0.12] 16.3%
H-GTEx −0.37 0.1576 −0.37 [−0.68; −0.06] 3.8%
H-UKBEC −0.29 0.1005 −0.29 [−0.48; −0.09] 9.4%
P-GTEx −0.14 0.1230 −0.14 [−0.38; 0.10] 6.3%
P-UKBEC −0.17 0.1210 −0.17 [−0.40; 0.07] 6.5%
Random effects model −0.19 [−0.25; −0.13] 100%
Heterogeneity: I-squared=0%, tau-squared=0, p=0.4617
−0.6 −0.4 −0.2 0 0.2 0.4 0.6
Beta
U
rs12491598 - RSRC1
Study TE seTE 95%-CI W(random)
C-GTEx 0.26 0.0872 0.26 [0.09; 0.43] 4.3%
C-UKBEC 0.06 0.0394 0.06 [−0.02; 0.14] 21.2%
F-GTEx 0.08 0.0721 0.08 [−0.07; 0.22] 6.3%
F-UKBEC 0.11 0.0390 0.11 [0.03; 0.19] 21.6%
H-GTEx 0.11 0.0684 0.11 [−0.02; 0.25] 7.0%
H-UKBEC 0.15 0.0428 0.15 [0.06; 0.23] 17.9%
P-GTEx 0.19 0.0950 0.19 [0.01; 0.38] 3.6%
P-UKBEC 0.09 0.0427 0.09 [0.01; 0.17] 18.1%
Random effects model 0.11 [0.07; 0.15] 100%
Heterogeneity: I-squared=0%, tau-squared=0, p=0.4583
−0.4 −0.2 0 0.2 0.4
Beta
V
rs832190 - AC136289.1
Study TE seTE 95%-CI W(random)
C-GTEx −0.03 0.1030 −0.03 [−0.23; 0.18] 2.4%
C-UKBEC −0.08 0.0400 −0.08 [−0.16; 0.00] 15.7%
F-GTEx −0.05 0.1001 −0.05 [−0.25; 0.15] 2.5%
F-UKBEC −0.08 0.0310 −0.08 [−0.14; −0.02] 26.0%
H-GTEx −0.30 0.1048 −0.30 [−0.51; −0.10] 2.3%
H-UKBEC −0.09 0.0301 −0.09 [−0.15; −0.03] 27.7%
P-GTEx −0.08 0.1231 −0.08 [−0.32; 0.16] 1.7%
P-UKBEC −0.11 0.0339 −0.11 [−0.18; −0.05] 21.8%
Random effects model −0.10 [−0.13; −0.06] 100%
Heterogeneity: I-squared=0%, tau-squared=0, p=0.6370
−0.4 −0.2 0 0.2 0.4
Beta
W
rs11779986 - DDHD2
Study TE seTE 95%-CI W(random)
C-GTEx −0.23 0.0572 −0.23 [−0.34; −0.12] 13.1%
C-UKBEC −0.07 0.0545 −0.07 [−0.18; 0.03] 14.4%
F-GTEx −0.09 0.0448 −0.09 [−0.18; 0.00] 20.8%
F-UKBEC −0.07 0.0693 −0.07 [−0.21; 0.06] 9.1%
H-GTEx −0.22 0.0731 −0.22 [−0.36; −0.08] 8.2%
H-UKBEC −0.14 0.0612 −0.14 [−0.26; −0.02] 11.5%
P-GTEx −0.14 0.0587 −0.14 [−0.25; −0.02] 12.5%
P-UKBEC −0.10 0.0644 −0.10 [−0.23; 0.02] 10.4%
Random effects model −0.13 [−0.17; −0.09] 100%
Heterogeneity: I-squared=3.7%, tau-squared=0.0001, p=0.4012
−0.3 −0.1 0 0.1 0.2 0.3
Beta
X
rs1233454 - ZNF155
Study TE seTE 95%-CI W(random)
C-GTEx 0.15 0.1217 0.15 [−0.09; 0.39] 2.2%
C-UKBEC 0.14 0.0329 0.14 [0.07; 0.20] 30.7%
F-GTEx −0.04 0.1392 −0.04 [−0.31; 0.24] 1.7%
F-UKBEC 0.08 0.0366 0.08 [0.01; 0.15] 24.9%
H-GTEx 0.03 0.1540 0.03 [−0.27; 0.34] 1.4%
H-UKBEC 0.11 0.0438 0.11 [0.02; 0.19] 17.3%
P-GTEx −0.17 0.1760 −0.17 [−0.52; 0.17] 1.1%
P-UKBEC 0.12 0.0402 0.12 [0.05; 0.20] 20.6%
Random effects model 0.11 [0.07; 0.14] 100%
Heterogeneity: I-squared=0%, tau-squared=0, p=0.6019
−0.4 −0.2 0 0.2 0.4
Beta

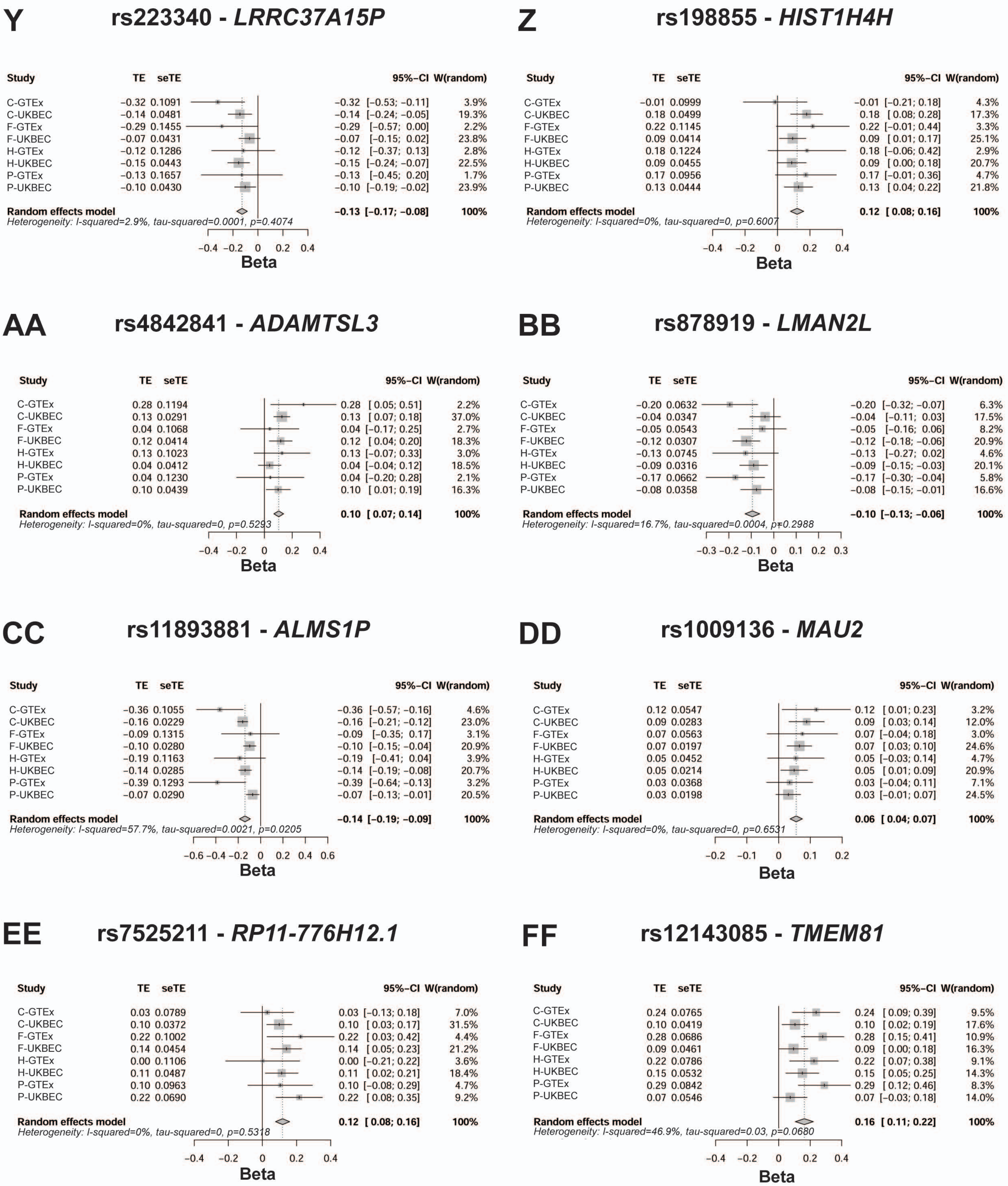
Y
rs223340 - LRRC37A15P
Study TE seTE 95%-CI W(random)
C-GTEx −0.32 0.1091 −0.32 [−0.53; −0.11] 3.9%
C-UKBEC −0.14 0.0481 −0.14 [−0.24; −0.05] 19.3%
F-GTEx −0.29 0.1455 −0.29 [−0.57; 0.00] 2.2%
F-UKBEC −0.07 0.0431 −0.07 [−0.15; 0.02] 23.8%
H-GTEx −0.12 0.1286 −0.12 [−0.37; 0.13] 2.8%
H-UKBEC −0.15 0.0443 −0.15 [−0.24; −0.07] 22.5%
P-GTEx −0.13 0.1657 −0.13 [−0.45; 0.20] 1.7%
P-UKBEC −0.10 0.0430 −0.10 [−0.19; −0.02] 23.9%
Random effects model −0.13 [−0.17; −0.08] 100%
Heterogeneity: I-squared=2.9%, tau-squared=0.0001, p=0.4074
−0.4 −0.2 0 0.2 0.4
Beta
Z
rs198855 - HIST1H4H
Study TE seTE 95%-CI W(random)
C-GTEx −0.01 0.0999 −0.01 [−0.21; 0.18] 4.3%
C-UKBEC 0.18 0.0499 0.18 [0.08; 0.28] 17.3%
F-GTEx 0.22 0.1145 0.22 [−0.01; 0.44] 3.3%
F-UKBEC 0.09 0.0414 0.09 [0.01; 0.17] 25.1%
H-GTEx 0.18 0.1224 0.18 [−0.06; 0.42] 2.9%
H-UKBEC 0.09 0.0455 0.09 [0.00; 0.18] 20.7%
P-GTEx 0.17 0.0956 0.17 [−0.01; 0.36] 4.7%
P-UKBEC 0.13 0.0444 0.13 [0.04; 0.22] 21.8%
Random effects model 0.12 [0.08; 0.16] 100%
Heterogeneity: I-squared=0%, tau-squared=0, p=0.6007
−0.4 −0.2 0 0.2 0.4
Beta
AA
rs4842841 - ADAMTSL3
Study TE seTE 95%-CI W(random)
C-GTEx 0.28 0.1194 0.28 [0.05; 0.51] 2.2%
C-UKBEC 0.13 0.0291 0.13 [0.07; 0.18] 37.0%
F-GTEx 0.04 0.1068 0.04 [−0.17; 0.25] 2.7%
F-UKBEC 0.12 0.0414 0.12 [0.04; 0.20] 18.3%
H-GTEx 0.13 0.1023 0.13 [−0.07; 0.33] 3.0%
H-UKBEC 0.04 0.0412 0.04 [−0.04; 0.12] 18.5%
P-GTEx 0.04 0.1230 0.04 [−0.20; 0.28] 2.1%
P-UKBEC 0.10 0.0439 0.10 [0.01; 0.19] 16.3%
Random effects model 0.10 [0.07; 0.14] 100%
Heterogeneity: I-squared=0%, tau-squared=0, p=0.5293
−0.4 −0.2 0 0.2 0.4
Beta
BB
rs878919 - LMAN2L
Study TE seTE 95%-CI W(random)
C-GTEx −0.20 0.0632 −0.20 [−0.32; −0.07] 6.3%
C-UKBEC −0.04 0.0347 −0.04 [−0.11; 0.03] 17.5%
F-GTEx −0.05 0.0543 −0.05 [−0.16; 0.06] 8.2%
F-UKBEC −0.12 0.0307 −0.12 [−0.18; −0.06] 20.9%
H-GTEx −0.13 0.0745 −0.13 [−0.27; 0.02] 4.6%
H-UKBEC −0.09 0.0316 −0.09 [−0.15; −0.03] 20.1%
P-GTEx −0.17 0.0662 −0.17 [−0.30; −0.04] 5.8%
P-UKBEC −0.08 0.0358 −0.08 [−0.15; −0.01] 16.6%
Random effects model −0.10 [−0.13; −0.06] 100%
Heterogeneity: I-squared=16.7%, tau-squared=0.0004, p=0.2988
−0.3 −0.2 −0.1 0 0.1 0.2 0.3
Beta
CC
rs11893881 - ALMS1P
Study TE seTE 95%-CI W(random)
C-GTEx −0.36 0.1055 −0.36 [−0.57; −0.16] 4.6%
C-UKBEC −0.16 0.0229 −0.16 [−0.21; −0.12] 23.0%
F-GTEx −0.09 0.1315 −0.09 [−0.35; 0.17] 3.1%
F-UKBEC −0.10 0.0280 −0.10 [−0.15; −0.04] 20.9%
H-GTEx −0.19 0.1163 −0.19 [−0.41; 0.04] 3.9%
H-UKBEC −0.14 0.0285 −0.14 [−0.19; −0.08] 20.7%
P-GTEx −0.39 0.1293 −0.39 [−0.64; −0.13] 3.2%
P-UKBEC −0.07 0.0290 −0.07 [−0.13; −0.01] 20.5%
Random effects model −0.14 [−0.19; −0.09] 100%
Heterogeneity: I-squared=57.7%, tau-squared=0.0021, p=0.0205
−0.6 −0.4 −0.2 0 0.2 0.4 0.6
Beta
DD
rs1009136 - MAU2
Study TE seTE 95%-CI W(random)
C-GTEx 0.12 0.0547 0.12 [0.01; 0.23] 3.2%
C-UKBEC 0.09 0.0283 0.09 [0.03; 0.14] 12.0%
F-GTEx 0.07 0.0563 0.07 [−0.04; 0.18] 3.0%
F-UKBEC 0.07 0.0197 0.07 [0.03; 0.10] 24.6%
H-GTEx 0.05 0.0452 0.05 [−0.03; 0.14] 4.7%
H-UKBEC 0.05 0.0214 0.05 [0.01; 0.09] 20.9%
P-GTEx 0.03 0.0368 0.03 [−0.04; 0.11] 7.1%
P-UKBEC 0.03 0.0198 0.03 [−0.01; 0.07] 24.5%
Random effects model 0.06 [0.04; 0.07] 100%
Heterogeneity: I-squared=0%, tau-squared=0, p=0.6531
−0.2 −0.1 0 0.1 0.2
Beta
EE
rs7525211 - RP11-776H12.1
Study TE seTE 95%-CI W(random)
C-GTEx 0.03 0.0789 0.03 [−0.13; 0.18] 7.0%
C-UKBEC 0.10 0.0372 0.10 [0.03; 0.17] 31.5%
F-GTEx 0.22 0.1002 0.22 [0.03; 0.42] 4.4%
F-UKBEC 0.14 0.0454 0.14 [0.05; 0.23] 21.2%
H-GTEx 0.00 0.1106 0.00 [−0.21; 0.22] 3.6%
H-UKBEC 0.11 0.0487 0.11 [0.02; 0.21] 18.4%
P-GTEx 0.10 0.0963 0.10 [−0.08; 0.29] 4.7%
P-UKBEC 0.22 0.0690 0.22 [0.08; 0.35] 9.2%
Random effects model 0.12 [0.08; 0.16] 100%
Heterogeneity: I-squared=0%, tau-squared=0, p=0.5318
−0.4 −0.2 0 0.2 0.4
Beta
FF
rs12143085 - TMEM81
Study TE seTE 95%-CI W(random)
C-GTEx 0.24 0.0765 0.24 [0.09; 0.39] 9.5%
C-UKBEC 0.10 0.0419 0.10 [0.02; 0.19] 17.6%
F-GTEx 0.28 0.0686 0.28 [0.15; 0.41] 10.9%
F-UKBEC 0.09 0.0461 0.09 [0.00; 0.18] 16.3%
H-GTEx 0.22 0.0786 0.22 [0.07; 0.38] 9.1%
H-UKBEC 0.15 0.0532 0.15 [0.05; 0.25] 14.3%
P-GTEx 0.29 0.0842 0.29 [0.12; 0.46] 8.3%
P-UKBEC 0.07 0.0546 0.07 [−0.03; 0.18] 14.0%
Random effects model 0.16 [0.11; 0.22] 100%
Heterogeneity: I-squared=46.9%, tau-squared=0.03, p=0.0680
−0.4 −0.2 0 0.2 0.4
Beta

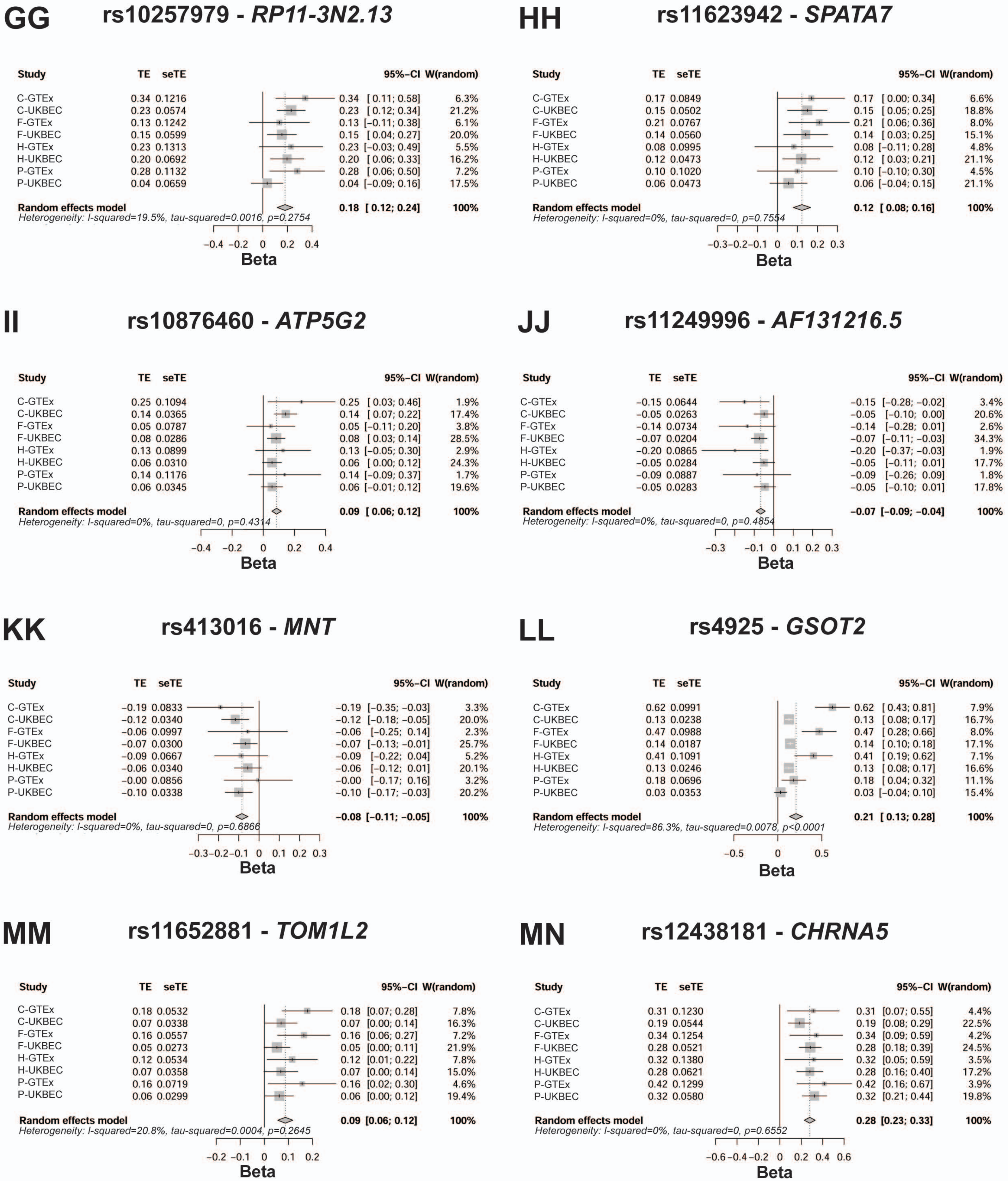
GG
rs10257979 - RP11-3N2.13
Study TE seTE 95%-CI W(random)
C-GTEx 0.34 0.1216 0.34 [0.11; 0.58] 6.3%
C-UKBEC 0.23 0.0574 0.23 [0.12; 0.34] 21.2%
F-GTEx 0.13 0.1242 0.13 [−0.11; 0.38] 6.1%
F-UKBEC 0.15 0.0599 0.15 [0.04; 0.27] 20.0%
H-GTEx 0.23 0.1313 0.23 [−0.03; 0.49] 5.5%
H-UKBEC 0.20 0.0692 0.20 [0.06; 0.33] 16.2%
P-GTEx 0.28 0.1132 0.28 [0.06; 0.50] 7.2%
P-UKBEC 0.04 0.0659 0.04 [−0.09; 0.16] 17.5%
Random effects model 0.18 [0.12; 0.24] 100%
Heterogeneity: I-squared=19.5%, tau-squared=0.0016, p=0.2754
−0.4 −0.2 0 0.2 0.4
Beta
HH
rs11623942 - SPATA7
Study TE seTE 95%-CI W(random)
C-GTEx 0.17 0.0849 0.17 [0.00; 0.34] 6.6%
C-UKBEC 0.15 0.0502 0.15 [0.05; 0.25] 18.8%
F-GTEx 0.21 0.0767 0.21 [0.06; 0.36] 8.0%
F-UKBEC 0.14 0.0560 0.14 [0.03; 0.25] 15.1%
H-GTEx 0.08 0.0995 0.08 [−0.11; 0.28] 4.8%
H-UKBEC 0.12 0.0473 0.12 [0.03; 0.21] 21.1%
P-GTEx 0.10 0.1020 0.10 [−0.10; 0.30] 4.5%
P-UKBEC 0.06 0.0473 0.06 [−0.04; 0.15] 21.1%
Random effects model 0.12 [0.08; 0.16] 100%
Heterogeneity: I-squared=0%, tau-squared=0, p=0.7554
−0.3 −0.2 −0.1 0 0.1 0.2 0.3
Beta
II
rs10876460 - ATP5G2
Study TE seTE 95%-CI W(random)
C-GTEx 0.25 0.1094 0.25 [0.03; 0.46] 1.9%
C-UKBEC 0.14 0.0365 0.14 [0.07; 0.22] 17.4%
F-GTEx 0.05 0.0787 0.05 [−0.11; 0.20] 3.8%
F-UKBEC 0.08 0.0286 0.08 [0.03; 0.14] 28.5%
H-GTEx 0.13 0.0899 0.13 [−0.05; 0.30] 2.9%
H-UKBEC 0.06 0.0310 0.06 [0.00; 0.12] 24.3%
P-GTEx 0.14 0.1176 0.14 [−0.09; 0.37] 1.7%
P-UKBEC 0.06 0.0345 0.06 [−0.01; 0.12] 19.6%
Random effects model 0.09 [0.06; 0.12] 100%
Heterogeneity: I-squared=0%, tau-squared=0, p=0.4314
−0.4 −0.2 0 0.2 0.4
Beta
JJ
rs11249996 - AF131216.5
Study TE seTE 95%-CI W(random)
C-GTEx −0.15 0.0644 −0.15 [−0.28; −0.02] 3.4%
C-UKBEC −0.05 0.0263 −0.05 [−0.10; 0.00] 20.6%
F-GTEx −0.14 0.0734 −0.14 [−0.28; 0.01] 2.6%
F-UKBEC −0.07 0.0204 −0.07 [−0.11; −0.03] 34.3%
H-GTEx −0.20 0.0865 −0.20 [−0.37; −0.03] 1.9%
H-UKBEC −0.05 0.0284 −0.05 [−0.11; 0.01] 17.7%
P-GTEx −0.09 0.0887 −0.09 [−0.26; 0.09] 1.8%
P-UKBEC −0.05 0.0283 −0.05 [−0.10; 0.01] 17.8%
Random effects model −0.07 [−0.09; −0.04] 100%
Heterogeneity: I-squared=0%, tau-squared=0, p=0.4854
−0.3 −0.1 0 0.1 0.2 0.3
Beta
KK
rs413016 - MNT
Study TE seTE 95%-CI W(random)
C-GTEx −0.19 0.0833 −0.19 [−0.35; −0.03] 3.3%
C-UKBEC −0.12 0.0340 −0.12 [−0.18; −0.05] 20.0%
F-GTEx −0.06 0.0997 −0.06 [−0.25; 0.14] 2.3%
F-UKBEC −0.07 0.0300 −0.07 [−0.13; −0.01] 25.7%
H-GTEx −0.09 0.0667 −0.09 [−0.22; 0.04] 5.2%
H-UKBEC −0.06 0.0340 −0.06 [−0.12; 0.01] 20.1%
P-GTEx −0.00 0.0856 −0.00 [−0.17; 0.16] 3.2%
P-UKBEC −0.10 0.0338 −0.10 [−0.17; −0.03] 20.2%
Random effects model −0.08 [−0.11; −0.05] 100%
Heterogeneity: I-squared=0%, tau-squared=0, p=0.6866
−0.3 −0.2 −0.1 0 0.1 0.2 0.3
Beta
LL
rs4925 - GSOT2
Study TE seTE 95%-CI W(random)
C-GTEx 0.62 0.0991 0.62 [0.43; 0.81] 7.9%
C-UKBEC 0.13 0.0238 0.13 [0.08; 0.17] 16.7%
F-GTEx 0.47 0.0988 0.47 [0.28; 0.66] 8.0%
F-UKBEC 0.14 0.0187 0.14 [0.10; 0.18] 17.1%
H-GTEx 0.41 0.1091 0.41 [0.19; 0.62] 7.1%
H-UKBEC 0.13 0.0246 0.13 [0.08; 0.17] 16.6%
P-GTEx 0.18 0.0696 0.18 [0.04; 0.32] 11.1%
P-UKBEC 0.03 0.0353 0.03 [−0.04; 0.10] 15.4%
Random effects model 0.21 [0.13; 0.28] 100%
Heterogeneity: I-squared=86.3%, tau-squared=0.0078, p<0.0001
−0.5 0 0.5
Beta
MM
rs11652881 - TOM1L2
Study TE seTE 95%-CI W(random)
C-GTEx 0.18 0.0532 0.18 [0.07; 0.28] 7.8%
C-UKBEC 0.07 0.0338 0.07 [0.00; 0.14] 16.3%
F-GTEx 0.16 0.0557 0.16 [0.06; 0.27] 7.2%
F-UKBEC 0.05 0.0273 0.05 [0.00; 0.11] 21.9%
H-GTEx 0.12 0.0534 0.12 [0.01; 0.22] 7.8%
H-UKBEC 0.07 0.0358 0.07 [0.00; 0.14] 15.0%
P-GTEx 0.16 0.0719 0.16 [0.02; 0.30] 4.6%
P-UKBEC 0.06 0.0299 0.06 [0.00; 0.12] 19.4%
Random effects model 0.09 [0.06; 0.12] 100%
Heterogeneity: I-squared=20.8%, tau-squared=0.0004, p=0.2645
−0.2 −0.1 0 0.1 0.2
Beta
MN
rs12438181 - CHRNA5
Study TE seTE 95%-CI W(random)
C-GTEx 0.31 0.1230 0.31 [0.07; 0.55] 4.4%
C-UKBEC 0.19 0.0544 0.19 [0.08; 0.29] 22.5%
F-GTEx 0.34 0.1254 0.34 [0.09; 0.59] 4.2%
F-UKBEC 0.28 0.0521 0.28 [0.18; 0.39] 24.5%
H-GTEx 0.32 0.1380 0.32 [0.05; 0.59] 3.5%
H-UKBEC 0.28 0.0621 0.28 [0.16; 0.40] 17.2%
P-GTEx 0.42 0.1299 0.42 [0.16; 0.67] 3.9%
P-UKBEC 0.32 0.0580 0.32 [0.21; 0.44] 19.8%
Random effects model 0.28 [0.23; 0.33] 100%
Heterogeneity: I-squared=0%, tau-squared=0, p=0.6552
−0.6 −0.4 −0.2 0 0.2 0.4 0.6
Beta

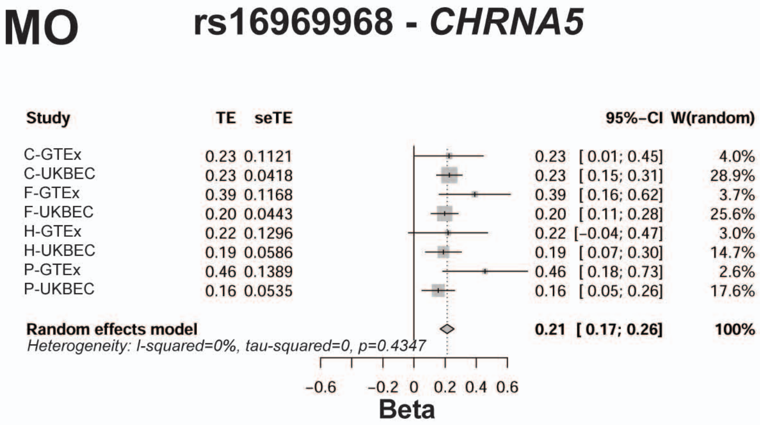
MO
rs16969968 - *CHRNA5*
Study TE seTE 95%-CI W(random)
C-GTEx 0.23 0.1121 0.23 [0.01; 0.45] 4.0%
C-UKBEC 0.23 0.0418 0.23 [0.15; 0.31] 28.9%
F-GTEx 0.39 0.1168 0.39 [0.16; 0.62] 3.7%
F-UKBEC 0.20 0.0443 0.20 [0.11; 0.28] 25.6%
H-GTEx 0.22 0.1296 0.22 [-0.04; 0.47] 3.0%
H-UKBEC 0.19 0.0586 0.19 [0.07; 0.30] 14.7%
P-GTEx 0.46 0.1389 0.46 [0.18; 0.73] 2.6%
P-UKBEC 0.16 0.0535 0.16 [0.05; 0.26] 17.6%
Random effects model 0.21 [0.17; 0.26] 100%
Heterogeneity: I-squared=0%, tau-squared=0, p=0.4347
−0.6 −0.2 0 0.2 0.4 0.6
Beta
