# Supplementary material for: Identification of expression quantitative trait loci associated with schizophrenia and affective disorders in normal brain tissue
Source: PLoS Genet. 2018 Aug 24;14(8):e1007607. doi: 10.1371/journal.pgen.1007607 (PMC6126875; doi:10.1371/journal.pgen.1007607)
Supplement: S1 Data — (PDF) [file pgen.1007607.s031.pdf]

UK Brain Expression Consortium (UKBEC) members and affiliations

Mina Ryten[1,2]
Michael E Weale[1]
John Hardy[2]
Karishma D'Sa[1,2]
Adaikalavan Ramasamy[1,2,3]
Daniah Trabzuni[2,4]
Sebastian Guelfi[2]
Juan A. Botia[2]
Jana Vandrovcova[2]
Colin Smith[5]
Robert Walker[5]

[1]Department of Medical & Molecular Genetics, King's College London, Guy's Hospital, London, UK

[2]Reta Lila Weston Research Laboratories, Department of Molecular Neuroscience, University College London (UCL) Institute of Neurology, London, UK

[3]Jenner Institute, University of Oxford, Oxford, UK

[4]Department of Genetics, King Faisal Specialist Hospital and Research Centre, Riyadh, Saudi Arabia

[5]Department of Neuropathology, MRC Sudden Death Brain Bank Project, University of Edinburgh, Edinburgh, UK
